# Picosecond carrier dynamics in InAs and GaAs revealed by ultrafast electron microscopy

**DOI:** 10.1126/sciadv.adn8980

**Published:** 2024-05-15

**Authors:** Christopher Perez, Scott R. Ellis, Francis M. Alcorn, Eric J. Smoll, Elliot J. Fuller, Francois Leonard, David Chandler, A. Alec Talin, Ravindra Singh Bisht, Shriram Ramanathan, Kenneth E. Goodson, Suhas Kumar

**Affiliations:** ^1^ Sandia National Laboratories, Livermore, CA, USA.; ^2^ Department of Mechanical Engineering, Stanford University, Stanford, CA, USA.; ^3^ Intel Corporation, San Jose, CA, USA.; ^4^ Department of Electrical and Computer Engineering, Rutgers, The State University of New Jersey, Piscataway, NJ, USA.

## Abstract

Understanding the limits of spatiotemporal carrier dynamics, especially in III-V semiconductors, is key to designing ultrafast and ultrasmall optoelectronic components. However, identifying such limits and the properties controlling them has been elusive. Here, using scanning ultrafast electron microscopy, in bulk n-GaAs and p-InAs, we simultaneously measure picosecond carrier dynamics along with three related quantities: subsurface band bending, above-surface vacuum potentials, and surface trap densities. We make two unexpected observations. First, we uncover a negative-time contrast in secondary electrons resulting from an interplay among these quantities. Second, despite dopant concentrations and surface state densities differing by many orders of magnitude between the two materials, their carrier dynamics, measured by photoexcited band bending and filling of surface states, occur at a seemingly common timescale of about 100 ps. This observation may indicate fundamental kinetic limits tied to a multitude of material and surface properties of optoelectronic III-V semiconductors and highlights the need for techniques that simultaneously measure electro-optical kinetic properties.

## Teaser

Picosecond-resolved scanning electron microscopy reveals photoexcited charge carrier dynamics in III-V semiconductors.

## INTRODUCTION

The excitation, transport, and recombination of charge carriers in III-V semiconductors ultimately determine their viability for numerous applications from photovoltaics to optoelectronics (*1*, *2*). For example, the performance of III-V multijunction photovoltaic cells depends on photogenerated carrier lifetimes, diffusion lengths, and the charge separation of the carriers (*3*). Semiconductors for fast (<300-ps rise time) photodetection rely on efficient charge separation and high carrier mobilities (*4*, *5*). Further, surface states can limit the tuning range of the field-effect devices based on III-V materials, complicating the design of on-off ratios and threshold or flat-band voltages (*6*). Broadly speaking, the macroscale performance of such devices have nanoscopic origins within dynamics that occur at ultrafast (sub-picosecond) timescales. Because carrier densities and surface trap states in semiconductors markedly change with dimensions or solid interfaces (*4*, *7*), local disorder (*8*), and surface states (*9*), a spatiotemporal understanding can inform rational device design principles of optical and electronic devices as they approach sub-2-nm features in the near future.

**Fig. 1.**
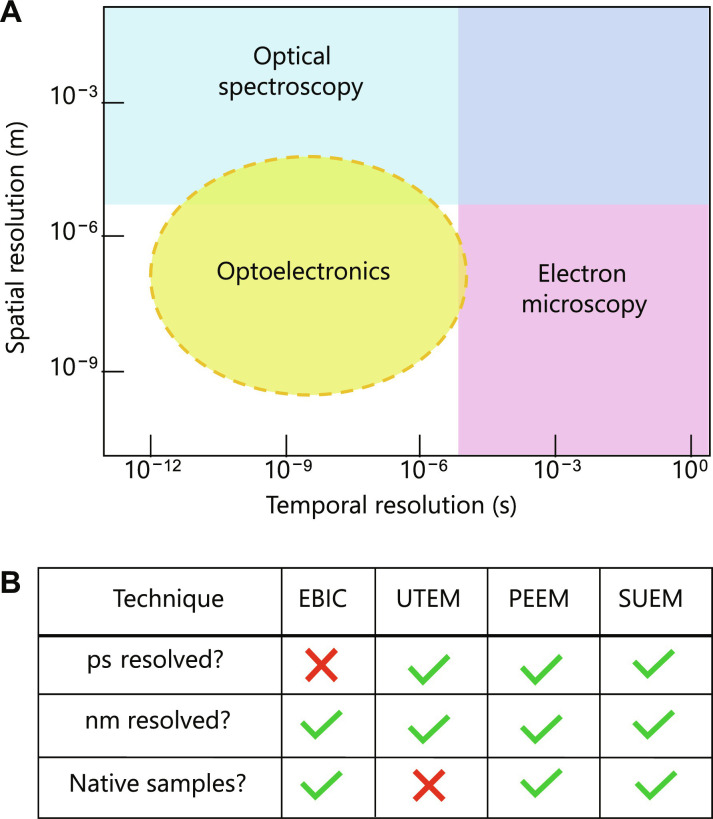
Comparison of electron microscopy based analytical techniques. (**A**) Optoelectronic devices operate in a spatiotemporal region that is difficult to reach by many conventional techniques. Electron microscopy can routinely image relevant structure on the micro/nano scale but lack the temporal resolution for resolving dynamics in these devices. Conversely optical spectroscopy can operate on these fast and ultrafast timescales but lack the submicron spatial resolution. (**B**) Various techniques have been developed to reach this spatiotemporal region with various advantages and disadvantages. SUEM offers the ability to resolve dynamics in native optoelectronic devices. EBIC, electron-beam–induced current; UTEM, ultrafast transmission electron microscopy; PEEM, photoemission electron microscopy; SUEM, scanning ultrafast electron microscopy.

Capturing the spatiotemporal evolution of excited carriers in semiconductors, however, is a considerable challenge that has been reasonably addressed by only a handful of techniques (Fig. 1) (*10*). Ultrafast transmission electron microscopy (UTEM) (*11*, *12*) has allowed the observation of metal-insulator phase transitions (*13*) and even spin crossover dynamics (*14*) but requires intensive sample preparation that yields non-native samples (i.e., not in a native operating device structure) of less than a few hundred nanometers in thickness. Time-resolved nonlinear photoemission electron microscopy can offer superior temporal resolution (*15*–*18*) but with a slight trade-off in spatial resolution (*19*). Conversely, scanning electron microscopy (SEM)–based techniques such as electron beam–induced current measurements can reveal notable phenomena in native state semiconductors with nanometer-scale spatial resolutions (*20*, *21*) but are limited to studies on timescales much slower than operational speeds of many practical devices. Thus, there is a gap in our understanding of the fundamental kinetic processes that govern optoelectronic devices and materials that rely on carrier dynamics, such as high-speed photodiodes. As an example, we do not understand whether and how fundamental speed limits to carrier dynamics depend on surface states, the speed with which we can fill such states and affect band bending potentials. Clearly, there is a need for a robust measurement technique that can nondestructively probe the spatiotemporal high-speed dynamics of excited carriers, along with the capability to measure defects and band potentials. Such a technique would enable prediction of device-level behavior without fully manufacturing and extensively measuring a device structure.

On the basis of the well-established SEM, scanning ultrafast electron microscopy (SUEM) quantifies the spatiotemporal evolution of carrier dynamics in semiconductors (*22*–*24*). SUEM combines the spatial resolution of SEM (sub-100 nm) with the resolution of ultrafast lasers (15 ps) in a stroboscopic fashion. Using modulated optical pump pulses, photoexcited carriers are created, which neutralize the native surface charge of a sample, decrease the degree of the electron band bending between the sample surface and the surrounding vacuum [i.e., space-charge region (SCR)], and detect its effects on the secondary electron (SE) counts. Because SUEM, like SEMs, relies on SE emission to detect charge dynamics, the SUEM SE contrast mechanism has been attributed to various factors ranging from variation in the subsurface band bending and work function to local carrier density and chemical composition (*25*–*27*). Identifying the contrast mechanisms contributing to the detection of altered SE counts remains elusive given observed phenomena such as anisotropic photocarrier diffusion (*28*), ballistic electron transport across p-n junctions (*29*), and super-diffusion of photoexcited carriers (*30*).

Here, we develop a novel implementation of SUEM using lock-in detection and examine the signals from the exposed semiconductor surfaces of n-GaAs and p-InAs. We find that, as the SEs drift through a SCR before they are emitted, there is an additional photoinduced contrast mechanism, the vacuum field effect, which differs from the well-documented subsurface effect. As such, we find that both the local surface potential and the potential in the vacuum at short distances above the surface dominate the SUEM signal. Together, these mechanisms allow simultaneous detection of local subsurface potentials, surface defect densities, and picosecond carrier dynamics. We find an abundance of surface trap states in both p-InAs and n-GaAs based on their photosaturation at high laser fluences (0.2 and 8 mJcm^−2^ in p-InAs and n-GaAs, respectively), and the dominance of the local surface potential in the SUEM signal implies that surface defects or traps govern the surface potential for these materials. Further, we noticed an unexpected common timescale for photosaturation in both materials (of about 100 ps), despite them having very different defect environments, dopant profiles, and bandgap energies. These insights place a fundamental limit on the timescales and the surface states of canonical III-V semiconductors, thereby providing a useful guideline for engineering optoelectronic materials.

### SUEM overview

The bulk single-crystal GaAs <100> and InAs <100> in our study were doped with Si and Zn to produce n-type and p-type materials, respectively (section S1). SUEM operates on a stroboscopic principle where a pulsed optical laser (pump) excites a sample, while a pulsed electron beam (probe) monitors carrier dynamics at the surface as a function of pump-probe time delay, detailed elsewhere (*31*, *32*). The probe is scanned across the sample to generate a SE contrast image at each time delay, thus forming an ultrafast SEM movie of the pump-induced carrier dynamics.

**Fig. 2.**
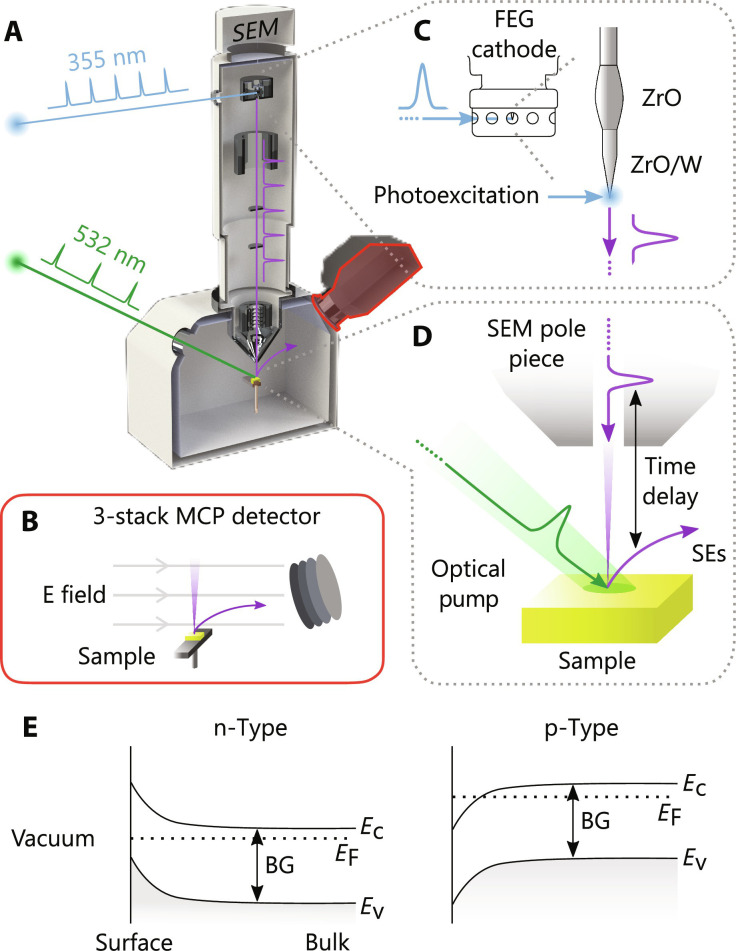
Schematic of the SUEM setup. (**A**) A simplified schematic of the SUEM setup consisting of a primary 1064-nm laser source that is split into pump and probe arms frequency doubled and tripled to 532 and 355 nm, respectively. The 532-nm pump arm passes through a mechanical delay stage to provide a time resolution of 15 ps as it is focused into the SEM chamber. The SEs are collected with a three-stack micro-channel plate (MCP) detector shown in (**B**). The 355-nm arm is focused on the tip of the field emitting gun (FEG) of the SEM and photoexcites pulses of electrons seen in (**C**). E field, electric field. Pump-probe data are then taken as a function of time delay between the laser pump and SE probe, as illustrated in (**D**). (**E**) Simplified bandgap diagrams for n- and p-type materials, showing the band bending at the surface/vacuum interface, conduction band energy (*E*_C_), valence band energy (*E*_V_), Fermi energy (*E*_F_), and bandgap (BG).

Our implementation of SUEM is illustrated in Fig. 2A as a simplified schematic. A preliminary version of the SUEM was described in our prior work (*33*). Here, a 1064-nm laser source (2-MHz repetition rate, 10-ps pulses) was coupled to a Philips-FEI XL30s SEM through optical ports that were built onto the SEM chamber and the field-emission gun. The SEs were collected with a three-stack micro-channel plate detector, illustrated in Fig. 2B, with a field above the sample to direct the electrons to the detector. Third-harmonic generation of the primary beam allowed us to generate 532-nm pump and 355-nm probe arms, with the pump directed toward the sample and the probe focused onto the apex of a ZrO/W Schottky field emitter to produce a pulsed electron beam, illustrated in Fig. 2C. The SE signal was then read by two lock-in amplifiers referencing 1 and 2 MHz originating from the laser source. The electron beam (with 7 kV of accelerating voltage) was rastered slowly such that a unique Fourier amplitude could evolve under each pixel. Three frames were averaged before progressing to the next pump delay position and measurements at time delays from −6 to 10 ns. The instrument response, yielding our time resolution of 15 ps, is a result of the cross correlation of the optical and electron pulses (*33*). Pump-probe data are then taken as a function of time delay between the laser pump and SE probe, illustrated in Fig. 2D. Further details regarding our SUEM instrument can be found in section S2 (fig. S1).

Changes in work function were measured separately using Kelvin probe force microscopy (KPFM) (*34*, *35*) performed in an inert argon environment. A 530-nm continuous-wave diode laser was focused over the probe tip, producing a fluence of approximately 5 kW cm^−2^. The laser was cycled on and off as the tip was scanned, and the corresponding change in contact potential difference was recorded. This change is directly related to the sample work function and is commonly referred to as the surface photovoltage (SPV). Additional KPFM details are provided in section S3 (fig. S2).

## RESULTS

### SE contrast theory

**Fig. 3.**
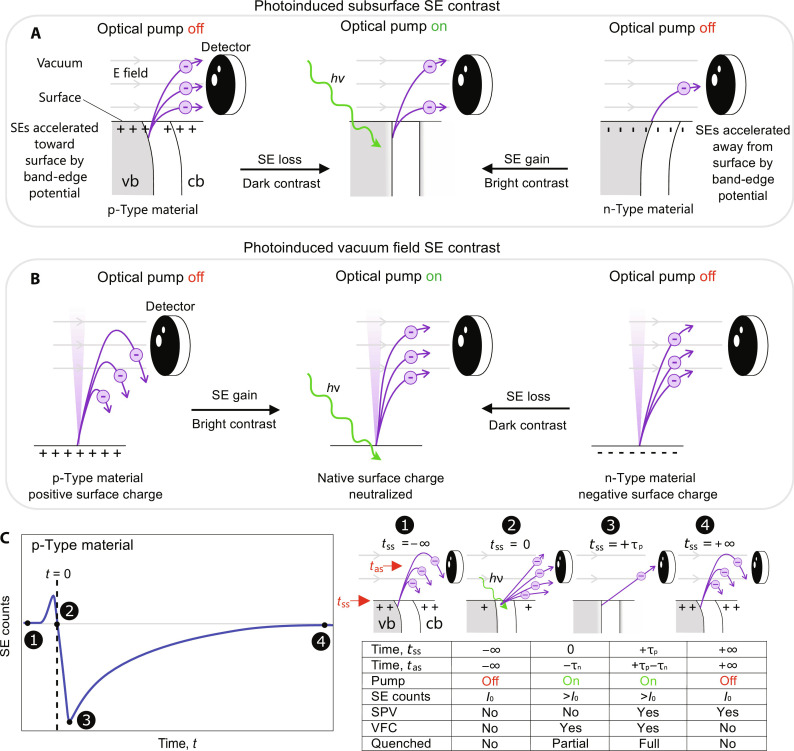
SUEM contrast mechanisms. The photoinduced contrast mechanisms of SE emission. These are conceptually separated into two parts: (**A**) subsurface processes, which describe the drift of SEs toward or away from the surface due to band bending, and (**B**) vacuum field processes, which occur when few emitted SEs travel from the surface to the detector due to an electric field and are influenced by the established surface charge upon illumination. Incoming light flattens the valence/conduction bands (vb/cb) and neutralizes the native surface charge. Further, p- and n-type materials exhibit opposite contrast due to their opposing native surface charges and band-edge curvatures. (**C**) The physical origin of SE contrast in a SUEM experiment at early negative time delays for p-type materials. Slowly moving SEs take time to reach the detector and are in transit even before photoexcitation. This results in a bright contrast at negative time delays, denoted as time above-surface (*t*_as_) with time values corresponding to time delay at which these SEs were generated, from the established SPV and vacuum field contrast (VFC) that accelerates them toward the detector. Electrons generated at positive time delays experience subsurface effects from photoexcitation, denoted by time subsurface (*t*_ss_). τ_p_ denotes a positive time delay for an electron pulse following photoexcitation, and τ_n_ denotes a negative time delay effect due to the VFC. The SE intensity symbols are arbitrary.

Our proposed SUEM contrast mechanism consists of photoinduced subsurface effects and vacuum field effects, as both change the photoinduced probability of SE detection generated by an electron beam pulse. The SUEM contrast also depends on the Fermi-level pinning at the surface-vacuum interface. The SUEM contrast mechanisms are illustrated in Fig. 2E. In routine SEM, contrast from subsurface effects (subsurface contrast) arises from the Fermi level of a semiconductor being pinned by localized surface states within its electronic bandgap, illustrated in Fig. 3A. Subsurface band bending exerts a drift force that affects the probability of SEs to diffuse toward and be emitted from the sample surface. For example, as SEs diffuse through the SCR in a p-type material, the downward bending (toward lower energies) of the band edge accelerates the SEs toward the surface and increases the electron escape depth inside the sample (originally ∼10 nm) (*36*) and results in the relatively bright contrast of p-doped materials commonly observed in SEM imaging of semiconductor materials. In SUEM, when the band bending is relaxed with the introduction of photoexcited carriers, a dark (loss) contrast signal arises (for a p-doped sample). The opposite is observed for an n-type material and thus results in a bright (gain) contrast signal under photoexcitation.

The vacuum field contrast, on the other hand, is a result of the SE trajectories in the vacuum above the sample surface, illustrated in Fig. 3B. As the flat-band condition is imposed, the number of SEs emitted will change due to the neutralization of electric fields. For instance, in a p-type material, some of the low energy SEs are recaptured by the positive surface charge and are not detected. With photoexcitation, however, the probability of SE detection is increased, producing a bright (gain) contrast (Fig. 3B). The opposite is true for an n-type material, that is, the neutralization of the negative surface charge results in a dark (loss) contrast. We note that the field lines can be curved at the peripheries of the photon-electron interaction region (PEIR), thus complicating this simple description.

The timescales at which both contrast mechanisms emerge reflect real physical processes and must be considered in our SUEM signals. For instance, subsurface contrast is thought to appear at ∼1 ps or the time that it takes for a SE to diffuse an electron escape depth (∼10 nm) to the surface. As such, the subsurface effects, to be discussed later, are much faster than our instrument response time, primarily determined by laser pulse duration (15 ps), and substantially contribute to dynamics only at time delays greater than +10 ps. Special consideration is given to the vacuum field contrast. Slowly moving SEs are in transit even before photoexcitation, whereas photoexcitation rapidly establishes a SPV, influencing the slowly moving SEs. Thus, photoexcitation influences SEs that were emitted even before the photoexcitation takes place, leading to negative-time contrast. This description is illustrated in Fig. 3C, where a bright negative-time contrast for a p-type material is generated from the established negative SPV that accelerates SEs toward the detector. As discussed in section S4, the detected signal is a combination of the subsurface and vacuum field contrast contributions convolved with their respective temporal distributions. The instrument response time and distribution-weighted average time of flight (65 ps) for generated SEs, with velocities on the order of 10^6^ m/s, to pass through the vicinity of the laser spot (∼100 μm), were used to deconvolve both effects on the subsurface and vacuum field contrast, respectively.

### The SUEM contrast mechanism

**Fig. 4.**
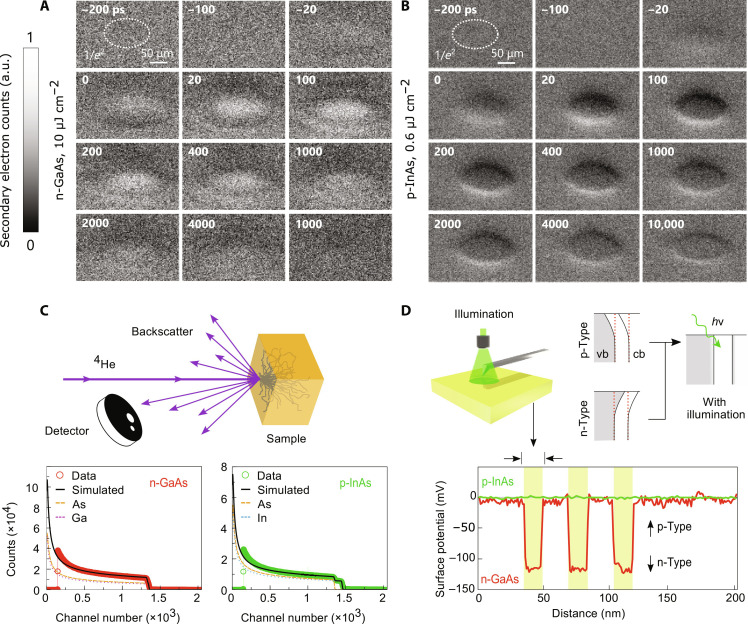
The SUEM image time series for n-GaAs and p-InAs. The SUEM image time series for (**A**) n-GaAs and (**B**) p-InAs. The peak fluences are shown in the time-series headers, while the beam 1/*e*^2^ radii are indicated with the dotted circles. The time delay is provided in the top left inset of each image. a.u., arbitrary units. (**C**) Illustration of Rutherford backscattering spectrometry (RBS) and RBS data for n-GaAs and p-InAs, showing the high stoichiometric quality of the samples. (**D**) The surface potentials upon 530-nm light cycling for n-GaAs and p-InAs measured with KPFM.

The SUEM images for n-GaAs and p-InAs as a function of time delay are displayed in Fig. 4 (A and B). The peak laser fluences for both materials were kept constant (10 and 0.6 μJ cm^−2^ for n-GaAs and p-InAs, respectively), although data were also taken at different fluences, provided in section S5 (figs. S3 and S4). Our hypothesized subsurface contrast mechanism (i.e., the flat-band condition) is upheld by the n-GaAs and p-InAs, exhibiting primarily bright and dark contrast, respectively. The contrast change is further quantified in section S5 (fig. S3), where the SE emission was compared for unmodulated SUEM images with the pump laser on and off. The compositions of the materials (Fig. 4C) were obtained using Rutherford backscattering spectrometry (RBS), showing a near-perfect 1:1 ratio for both Ga:As and In:As, indicating near-perfect stoichiometry of the samples (see section S6 and fig. S6) and minimal surface oxidation, consistent with prior results (*37*). KPFM data are also displayed in Fig. 4D and are in qualitative agreement with our SUEM data, yielding a SPV of ∼120 mV for n-GaAs. No change was detected for p-InAs. Small changes in SPV with illumination can be difficult to resolve in KPFM (*34*, *35*), especially given the substantially smaller bandgap of InAs (about 0.35 eV, compared to about 1.4 eV of GaAs). The null measurement in InAs, which will be revisited later, shows the need for understanding of key material parameters via kinetic properties (e.g., via SUEM) instead of via static properties.

The contrast inversion at the edges of the PEIR (top and bottom of the spatial features) can be explained by the vacuum field mechanism that depends on SE trajectories. We suspect that the electrostatic environment within our SEM chamber is such that divergent field lines form around the periphery of the probed region and result in the observed effect. For instance, the differential negative charge of the PEIR region accelerates SEs emitted from the bottom of the image toward the detector, while those emitted from the top of the image are accelerated away from the detector, which is most obvious in Fig. 4B for p-InAs (see section S7). This effect was also observed using time-resolved x-ray photoelectron spectroscopy (*38*) and has not yet been identified in SUEM experiments, with more discussion provided in section S4.

Contrast changes observed in our experiments differ from previous studies on GaAs (*39*, *40*) at both negative and positive time delays, reflecting differences in experimental parameters that affect SE generation and photo-responses. Prior studies have observed decreased electron contrast at both negative and positive time delays, whereas, here, we observe an inverted contrast change from negative to positive time delays and increased SE contrast due to photoexcitation. Our experiments were performed with a smaller primary beam energy than prior studies (7 keV compared to prior studies that used 30 keV), resulting in shallower SE generation depths, and with laser pulses of 15 ps, which are much longer than the SE escape time (∼1 ps). Consequently, subsurface effects that were suggested to result in SUEM signal at negative time delays in prior studies (*39*) can be ignored at negative time delays in our results. Instead, SUEM contrast at negative time delays in our studies result from the above-surface vacuum field effect, which affect SE contrast in an opposite way compared to subsurface band bending effects. This process results in an inversion in SE contrast from negative to positive time delays. In addition, considerations should be made for different working definitions for time zero between experiments, which will affect consideration of what groups of electrons constitute signals at negative versus positive time delays. Proliferation of SUEM research would benefit from a more established definition of time zero.

The difference in SE contrast at positive time delays is less obvious and may reflect differences due to SE escape depths, as shallower SE generation reduces the probability of scattering events that can relax photoexcited electrons (*39*). However, SUEM contrast of GaAs is also known to be affected by surface structure (*40*), confounding contrast analysis at these positive time delays. Regardless, SUEM contrast at time delays greater than ∼10 ps is necessarily due to subsurface mechanisms.

### Photosaturation and surface state density

**Fig. 5.**
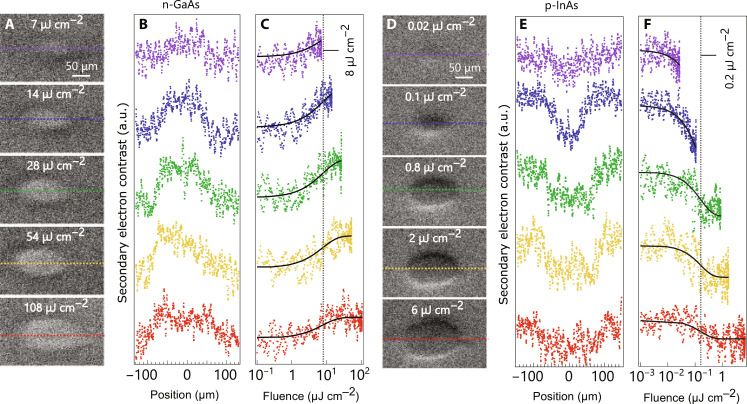
Fluence dependence of the SUEM signal. Fluence dependence of the SUEM signal from (**A** to **C**) n-GaAs and (**D** to **F**) p-InAs at a constant pump-probe time delay of 281 ps. The spatial profile of the SUEM signal was extracted over the dotted lines shown in (B) for n-GaAs and (E) for p-InAs. We note that the signal intensities have been shifted vertically, but their scaling is unchanged. The calculated fluence dependence is shown in (C) for n-GaAs and (F) for p-InAs, where a saturation behavior is observed. The gray vertical dashed lines indicate the fitting results of the form *C*(1 − *e*^−*f/f*s^), where *f* is the fluence and *f*_s_ is the fitted 1/*e* from saturation levels of 8.0 and 0.2 μJ cm^−2^ for n-GaAs and p-InAs, respectively.

The photoinduced SE contrast in our SUEM experiments is displayed in Fig. 5 for both n-GaAs and p-InAs at a suitable pump-probe time delay. The time delay was chosen to be 281 ps because this delay both provided a good signal and was slightly after the sharp peak of the signal at about 100 ps (will be detailed in the following section), thereby avoiding large perturbations associated with the signal’s sharp peak. By this time, most of the minority carriers have encountered the sample-vacuum interface, having had the opportunity to become trapped by the surface potential given the strong absorption of both materials (*25*–*27*). With stronger absorption, carriers are generated closer to the surface and require less time to become traps. Thus, this time delay provided a good starting point to observe recombination dynamics (see section S8). The SUEM signals were extracted over cross sections of the PEIR, illustrated as dotted horizontal lines, and are displayed as functions of position and fluence. The fluence itself was inferred from the measured beam profile to convert between spatial information to SE intensity dependence at each position (see section S9).

Our data reveal saturation behavior when subjecting n-GaAs and p-InAs to varied laser fluences, above which the addition of photoexcited carriers has no effect on SE emission. To determine the 1/*e* saturation fluence (*f*_sat_), we take the SE contrast as a function of fluence seen in Fig. 5 (C and F) and fit the fluence curves to an expression of the form *C*(1 − *e   *^*f*/*f*_sat_^). *e* is the Euler's number equal to 2.71828. This exercise results in saturation fluences of 8.0 and 0.2 μJ cm^−2^ for in n-GaAs and p-InAs, respectively, displayed as gray vertical dashed lines in Fig. 5 (C and F).

**Fig. 6.**
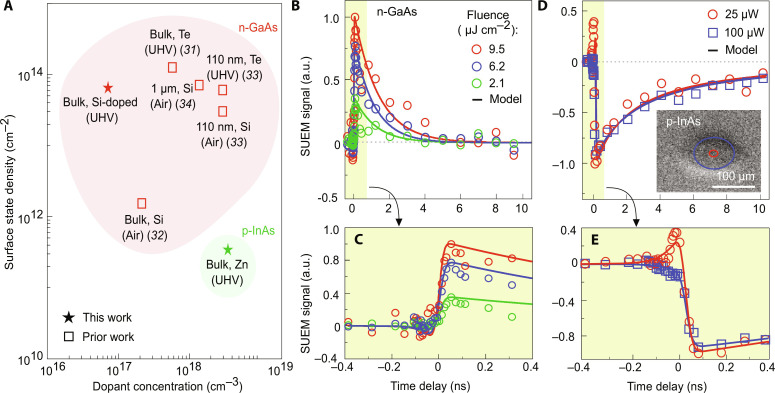
Comparison of dynamics in GaAS and InAs. (**A**) A benchmark plot with the various surface state densities with respect to dopant concentration found in the literature (*41*–*44*). The labels include the dopant type, the form factor of the sample, and the measurement conditions in air or ultrahigh vacuum (UHV). (**B**) SUEM data for n-GaAs at various laser fluences of 9.5, 6.2, and 2.1 μJ cm^−2^ in red, blue, and green, respectively. The solid lines are fits to the instrument response with initial charge carrier densities (ρ_cc_) of 25.4 × 10^12^ cm^−2^, 16.6 × 10^12^ cm^−2^, and 5.7 × 10^12^ cm^−2^. (**C**) An enlarged view of the time-resolved SUEM signal from −0.4 to 0.4 ns. (**D**) SUEM images of p-InAs indicating the red and blue elliptical regions, where fluences of 0.6 μJ cm^−2^ were obtained with laser powers of 25 and 100 μW in red and blue, respectively. The extracted time-resolved SUEM signal is displayed and the solid lines are fits to the instrument response with an initial ρ_cc_ of 1.5 × 10^12^ cm^−2^, as detailed in section S4. (**E**) An enlarged view of the time-resolved SUEM signal from −0.4 to 0.4 ns.

The analysis sheds light on electro-optic device limitations by yielding insights into the area density of absorbed photons at saturation due to full occupation of surface trap states. As mentioned previously, the data in Fig. 5 represent an evolved system where the area density of absorbed photons at saturation can be directly related to the surface state density [ρ_sat_(*h*ν)^−1^]. These densities were found the be 2 × 10^13^ for n-GaAs and 5 × 10^11^ cm^−2^ for p-InAs. The value for GaAs is in good agreement with those determined from other techniques in the literature (*41*–*44*), seen in Fig. 6A, while quantitative surface state density of bulk p-InAs is lacking in the literature (*45*). The high saturation fluence required for both materials, despite very different dopant profiles, occurs within a common timescale of roughly 100 ps. Further, these data were obtained by probing transient surface states at saturation, a condition not generally accessible with steady state techniques. Such steady state techniques often require passivation or ohmic contact to the sample (e.g., capacitive voltage). With continuous laser excitation (or other steady-state techniques), a dangerously high fluence of many GW cm^−2^ would be required to yield the high level of occupation of surface states seen in Fig. 5. This argument is similar to why static techniques such as KPFM are unsuitable for a wide variety of materials with different properties and explains the lack of signal in our KPFM measurements in InAs (Fig. 4D).

### Charge carrier and surface state dynamics

We present the time-resolved SUEM dynamics for n-GaAs and p-InAs in Fig. 6 (B and C). Figure 6B displays the n-GaAs temporal SUEM signal at different fluences. Figure 6D displays the p-InAs SUEM signal extracted in annular elliptical regions such that the temporal response to the fluence level of 0.2 μJ cm^−2^ could be compared. We find that the carrier dynamics are similar with rise times on the order of 100 ps and decay times on the order of 10 ns despite different laser powers and elliptical radii, indicating that fluence is the most important factor affecting the time dependence of our SUEM signal. This can also be seen in Fig. 5, where low fluence regions were observed to produce a relatively strong SE contrast compared to the high fluence regions. Unexpectedly, the PEIR can extend beyond the 1/*e*^2^ beam radius if the laser power is set sufficiently high without requiring lateral carrier diffusion as claimed in prior works (*30*). As discussed shortly, signal rise is due to diffusion of photoexcited charge carriers to materials surfaces, and decay is due to subsequent carrier recombination.

In our recent work (*33*), we have shown that the material response is well reproduced by modeling the one-dimensional (1D) diffusion of charge carriers near a surface treated as a saturable adsorption boundary condition (details provided in section S10). As such, we can consider surface traps forming a density of photoexcited charge carriers ρ_cc_(*t*, *z*) where *z* is in the vertical direction, to prepare an initial condition of

$$\rho_{\text{cc}}\left(0,Z\right)=\frac{f}{h\nu }e^{-\alpha z}$$ (1)

where *f*, ν, and α are the fluence, frequency of light, and attenuation coefficient, respectively, albeit the exponential term is not applied in these analyses. The charge carrier density evolves according to the equation

$$\frac{\partial \rho_{\text{cc}}\left(t,z\right)}{\partial t}=D_{\text{cc}}\frac{\partial^{2}\rho_{e}\left(t,z\right)}{\partial z^{2}}$$ (2)

where *D*_cc_ is the charge carrier diffusivity. We assume that that vertical gradients in carrier density are dominant because the lateral variations in carrier density are on the order of the 1/*e*^2^ beam radius in *y* (47 μm), while the gradients in *z* vary on the order of the absorption depth, approximately 30 and 15 nm for p-InAs and n-GaAs, respectively (*6*, *46*). We also consider a Langmuir adsorption dynamic equilibrium that exists between the free carriers at the surface and the vacant/occupied traps (*47*). We further ignore drift because we assume a flat-band condition, illustrated in Fig. 2. The best fit of the 1D diffusion model is displayed in Fig. 6 (B to D).

## DISCUSSION

The 1D diffusion model at a saturable surface yields quantitative dynamical insights. The initial rise and rapid decay of both materials are governed by electrons diffusing toward and away from the surface. Unexpectedly, the maximum contrast in the SUEM signal occurs within ∼100 ps in both p-InAs and n-GaAs. After the carrier density gradients have sufficiently flattened (corresponding to the maximum SUEM contrast), however, the dynamics are then governed by the recombination of traps with a characteristic time constant. This produces a stretched non-exponential decay that poses a more severe bandwidth limit relevant for multi-pulse applications, roughly ∼8 ns for both materials, which agrees favorably with the literature (*46*). We emphasize that the speed limits highlighted here are those of bulk materials with no clever use of thin films (*33*) or strategically arranged p-n junctions (*29*). Nonetheless, we provide the means to simultaneously and nondestructively characterize the fundamental transport limits in terms of carrier recombination behavior, surface diffusion limits, and the speed at which traps are filled.

The negative time delay behavior apparent in Fig. 6 (B to D) in both materials lends validity to our proposed vacuum field contrast mechanism. Having been attributed to the creation of internal SEs that lose excess energy as they travel toward and escape from the sample surface (*39*), negative time constants are difficult to explain when there is pump-probe interaction in vacuum. As discussed further in section S10, the timescale for photoexcited electrons to escape from the (∼50 nm) surface layer is less than 1 ps in both semiconductors and is far shorter than our instrument response time of 15 ps. Thus, a negative time constant of greater than 1 ps cannot be attributed to subsurface scattering effects and must arise from the SE transit in vacuum. This reasoning is further confirmed by our model fits in Fig. 6 (B and C), which consider the time of flight for a SE to overcome the influence of the SPV fields, demonstrating the robustness and applicability of our proposed SUEM contrast mechanisms.

Timescales for excitation and relaxation of GaAs measured in these studies are similar to prior SUEM studies on GaAs, at about 100 ps to 1 and 10 ns, respectively (*39*, *40*). Minor differences are expected due to differences in dopant profiles and surface composition. Thus, the agreement between these studies and the results presented here solidify SUEM as a tool for benchmarking fundamental speeds in optoelectronic devices.

Last, we reemphasize the interesting observation of both n-GaAs and p-InAs exhibiting similar timescales for photosaturation despite having vastly different dopant densities and surface state densities. The unexpected similarity in the kinetics may reflect differences in minority carrier mobilities and concentrations, as minor carriers are responsible for the observed SPV effects. Carrier mobilities, both electron and hole, are much higher for InAs than GaAs, but the minority carrier (hole) concentrations are larger for the lightly doped GaAs than the strongly doped InAs. The differences in carrier mobilities and concentrations could thus offset each other and may account for the similarity in timescales of dynamics observed in these studies. Other material properties will also affect these dynamics, most notably surface structure (*17*, *33*, *48*–*51*), which will have effects on other processes such as Fermi-level pinning. Such differences likely account for the observed differences in the contrast mechanisms and the dynamics between various SUEM studies on GaAs (*39*, *40*). Consequently, further studies are needed to correlate bulk and surface properties to optoelectronic properties with these materials. Studies of bulk parameters and surface states is an area of research where the SUEM is likely to find ample impact due to the ubiquity of SEM in materials research and minimal sample preparation requirements. Particularly, the SUEM is highly sensitive to surface potentials, while its insensitivity to passive surface coatings can be used to probe deep-buried bulk properties that affect surface potentials (*33*).

We elucidate the contrast mechanisms that underlie a novel implementation of the SUEM technique. Through calibrations using bulk n-GaAs, a well-established III-V semiconductor, we find that our measurements are dominated by both subsurface and vacuum field potentials. We then simultaneously determine the charge carrier dynamics and surface state densities for the first time in bulk p-InAs. Unexpectedly, in both p-InAs and n-GaAs, despite very different dopant densities and surface state densities, there appears to be similarity in the timescales for various processes: affecting the vacuum fields, imposing a flat band via optical excitation and relaxation of the excitation. Our dynamical data further provide fundamental limits to carrier transport relevant for single-shot high bandwidth and multi-pulse applications that invoke photosaturation and relaxation for bulk GaAs and InAs devices. Specifically, we isolate a negative-time contrast, which illustrates an interplay between vacuum field effects and relatively slower movement of electrons emitted from a surface. In addition, we substantiate a noncontact probe for investigating charge dynamics with surface sensitivity on the order of the electron band bending region at the surface (∼10 nm) (*36*), which can offer the link between the time and defect-limited electronic structure of materials. Such an understanding can provide exciting insights to guide the design of optoelectronic technologies with high commercial and environmental impact for emerging areas in sensing, computing, and energy conversion.

## MATERIALS AND METHODS

### Material growth and storage

The III-V materials used in this work were single-crystal n-type GaAs and p-type InAs purchased from MTI Corp. The n-GaAs was vertical gradient freeze grown (100) and Si-doped with a concentration of *N*_d_ = 3.8 × 10^16^ cm^−3^ to 6.2 × 10^16^ cm^−3^. The p-InAs was liquid encapsulated Czochralski grown (100) and Zn-doped with a concentration of *N*_a_ = 3.6 × 10^18^ cm^−3^. Both samples were used as-received and were stored in a nitrogen environment before SUEM characterization.

### Measurement of beam profile

The laser was measured in the sample chamber at the point of focus with a beam profiler (Thorlabs BP109-IR2) mounted such that it was normal to the electron beam, as seen in fig. S6. This positioning was intended to capture the angle of incidence of the pump beam. Measurements with the beam profiler were performed immediately after SUEM experiments, and the reflectivity of the p-polarized laser on the sample surface was measured to be less than 5%.

## Supplementary Materials

### This PDF file includes:

Sections S1 to S12 Figs. S1 to S10 References
