# Supplementary material for: Picosecond carrier dynamics in InAs and GaAs revealed by ultrafast electron microscopy
Source: Sci Adv. 2024 May 15;10(20):eadn8980. doi: 10.1126/sciadv.adn8980 (PMC11095486; doi:10.1126/sciadv.adn8980)
Supplement: Supplementary file 1 — Sections S1 to S12 Figs. S1 to S10 References [file sciadv.adn8980_sm.pdf]

Science Advances
AAAS

Supplementary Materials for

Picosecond carrier dynamics in InAs and GaAs revealed by ultrafast electron microscopy

Christopher Perez *et al.*

Corresponding author: Christopher Perez, cprez@alumni.stanford.edu; Francis M. Alcorn, fmalcor@sandia.gov; Suhas Kumar, su1@alumni.stanford.edu

Sci. Adv. **10**, eadn8980 (2024)
DOI: 10.1126/sciadv.adn8980

This PDF file includes:

Sections S1 to S12
Figs. S1 to S10
References

SECTIONS

S1. Sample details

S2. Implementation of scanning ultrafast electron microscopy (SUEM)

S3. Kelvin probe microscopy (KPM)

S4. Treatment of the SUEM signal at negative time delays

S5. Light-induced secondary electron loss and gain

S6. Rutherford backscattering spectrometry

S7. Estimation of the electrostatic environment

S8. Fluence saturation dependence in p-InAs and n-GaAs

S9. Measurement of beam profile

S10. Carrier diffusion and interfacial trapping model

S11. Comparison of SUEM signals at different laser powers

S12. Analysis of second moment contrast

S1. Sample details

The III-V materials used in this work were single crystal n-type GaAs and p-type InAs purchased from MTI. The n-GaAs was vertical gradient freeze (VGF) grown (100) and Si-doped with a concentration of $N_d = 3.8 - 6.2\times10^{16}$ cm^{-3}. The p-InAs was liquid encapsulated Czochralski (LEC) grown (100) and Zn-doped with a concentration of $N_a = 3.6\times10^{18}$ cm^{-3}. Both samples were used as-received and were stored in a nitrogen environment before SUEM characterization.

S2. Implementation of scanning ultrafast electron microscopy (SUEM)

A more complete schematic of our SUEM setup than that of Fig. 1 in the main text is shown in Fig. S1A and is based on designs reported in prior works (*31,32*). The secondary electrons (SEs) were collected with a 3-stack microchannel plate detector (MCP, Del Mar Photonics, 25 mm diameter) positioned 6 cm from the sample, shown schematically in Fig. S1B. The sample was biased to -350 V to repel the emitted SEs and maximize SE collection at the detector. The front face of the detector was biased to +300 V, with an additional +1000 V applied across each of the three MCPs. The current from the detector anode (+4700 V) was sent to a fast transimpedance current amplifier (Edmund Optics variable high speed current amplifier) with 10^4 gain.

Our experiment used a pulsed electron beam that was generated from 355 nm (120 mW) laser light (Fianium Hylase, fiber laser) that was focused on a cooled SEM field emitting gun (FEG), producing ~1 nA of current at the sample with a 300 μm column aperture, shown in Fig. S1C. The lens to focus the probe beam towards the FEG tip was precisely controlled using a 3-axis mirror

(3AM). Active laser stabilization was achieved by monitoring the pump/probe beams using beamsplitters (BSs) directed towards CCD cameras that worked in concert with motorized mirrors (MMs). The signal was then read by two lock-in amplifiers (Stanford Research Systems SR865A and SR865) referencing 1 MHz and 2 MHz, with integration times of 100 and 30 μs, respectively. To produce the reference signals, the repetition rate of the primary laser (2 MHz) was referenced by one of two lock-in amplifiers. The 2 MHz reference was also frequency divided (FD) shot to shot to produced a 1 MHz phase stable reference sent to the second lock-in amplifier and also an electro-optic modulator (EOM) acting on the 532 nm pump laser. The 2 MHz component of secondary electron signal reports on the integrated SE emission while 1 MHz component reports on the contrast in SE emission with and without the pump laser on the sample. The electron beam (7 kV accelerating voltage) was rastered slowly with a pixel dwell time of 156 μs and 367,930 pixels per frame such that a unique Fourier amplitude could evolve under each pixel. Three frames were averaged before progressing to the next pump delay position and measurements at time delays from -6 ns to 10 ns were taken. The instrument response of 14 ps is given by the cross correlation of the optical and electron pulses (*33*).

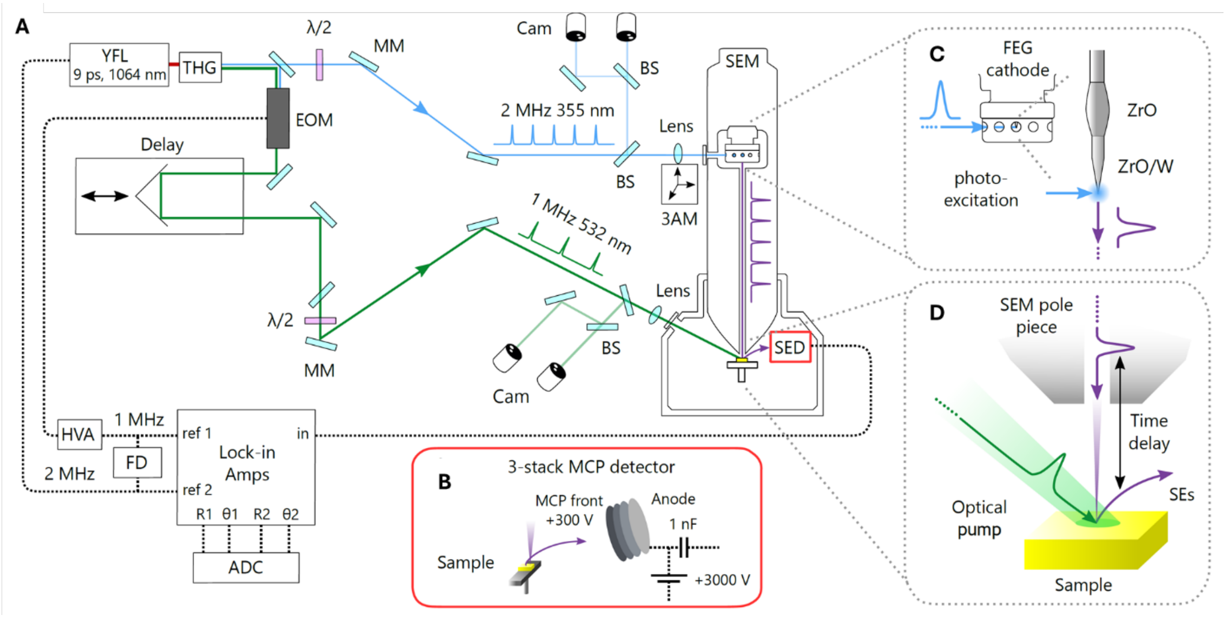

Figure S1. Detailed schematic of the SUEM setup. (**A**) A schematic of the SUEM setup consisting of a primary 1064 nm laser source that is split into pump and probe arms frequency doubled and tripled to 532 nm and 355 nm, respectively. The 532 nm pump arm passes through a mechanical delay stage to provide a time resolution on the order of 15 ps as it is focused into the SUEM chamber using a motorized mirror (MM). The 355 nm probe arm is focused onto the field emitting gun of the secondary electron microscope (SEM) using a 3-axis mirror (3AM). The secondary electron detector (SED) implemented in this work comprises of a 3-stack microchannel plate (MCP) detector shown in (**B**). The 355 nm arm is focused on the tip of the field emitting gun (FEG) of the SUEM and photo-excites pulses of electron emission, seen in

(**C**). Pump-probe data is then taken as a function of time delay between the laser pump and SE collection, illustrated in (**D**).

To align and focus the pump laser to the center of the SEM field of view, a 3 mm × 3 mm p47 phosphor plate (Kimball Physics) was mounted adjacent to the sample. The scintillation of the electron beam and multi-photon photoluminescence of the pump laser could be viewed by a microscope camera positioned behind the final pump turning mirror. An additional narrow bandpass 455 nm filter was inserted in front of the camera to isolate and observe the emission from the phosphor. To focus the pump laser on the sample surface, the laser was attenuated until the photoluminescence could be detected by the camera. A focusing lens was then adjusted to maximize the multiphoton photoluminescence at the sample surface.

S3. Kelvin probe microscopy (KPM)

As mentioned in the main text, KPM (Bruker ICON) was used to image the surface of both the n-GaAs and p-InAs with and without illumination by 530 nm light to measure the change in work function and verify the contrast mechanism observed with SUEM. Briefly, KPM is a non-contact atomic force microscopy technique that has shown utility in measuring the change in work function of semiconductor samples under illumination (*34,35*). All measurements were carried out in an inert argon environment (<1 ppm O2, <1ppm H2O) to remove any adsorbed water layers from the sample surface. A frequency modulated (FM) mode was utilized to improve spatial resolution, which approaches the probe tip radius of 50 nm. An Al-Si-Au calibration sample was used to verify the KPM measurement. Figure S2A shows how we decouple the topographical and contact potential difference (CPD) information in the resulting KPM surface scans. A horizontal linescan average of the CPD data is plotted in Fig. S2B, showing a clear difference in the CPD across the Al-Si-Au regions.

To collect KPM data under illumination, an optical access probe (OTESPA-R3) was utilized in order to allow top-down illumination of the sample without interference from the AFM cantilever. A 480 mW continuous wave 530 nm LED laser (Thorlabs M530L4) was focused to an approximately 100 µm × 100 µm spot about the probe tip, shown schematically in Fig. S2C. The incident fluence of the laser was estimated to be 5 kW cm^{-2} at the surface. The 520 nm light source was cycled on and off during the KPM measurement to note changes in the CPD, illustrated in Fig. S2D and Fig. S2E for n-GaAs and p-InAs, respectively. Similar to the Al-Si-Au calibration sample, these images were horizontally averaged to produce the 1D plot of surface potential and image distance in the main text.

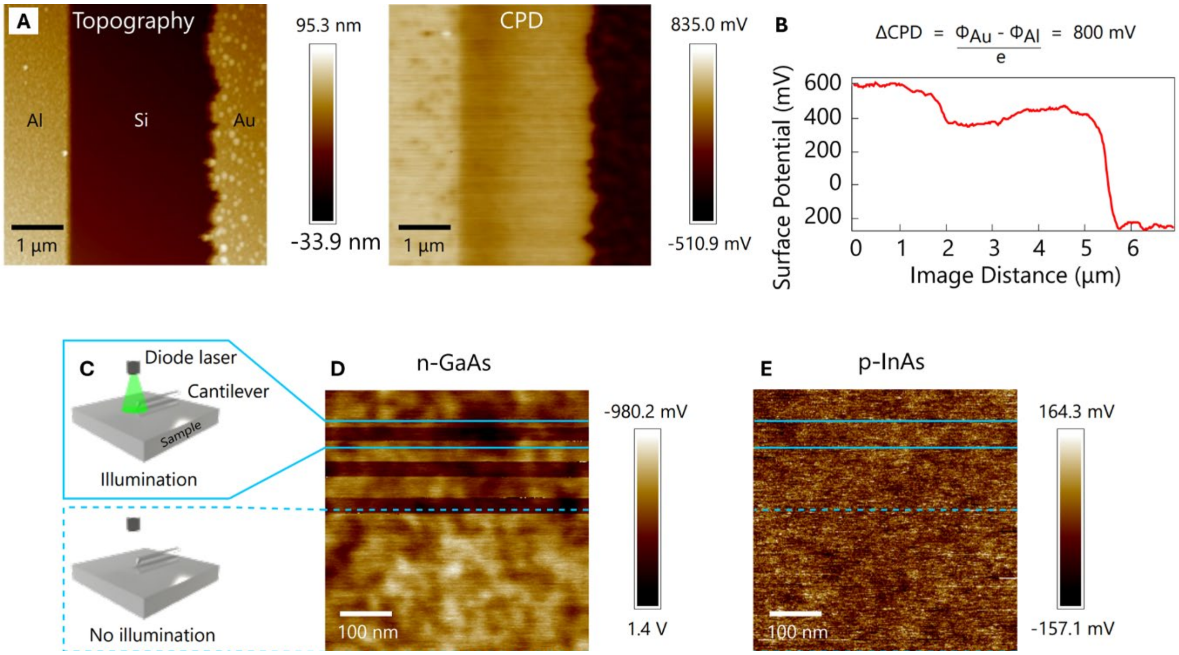

Figure S2. Kelvin probe microscopy on the samples measured in the SUEM. (**A**) The Al-Si-Au sample used to calibrate the Kelvin probe microscopy (KPM) measurement. Both the topography and surface potential data are shown, demonstrating our ability to decouple both types of information. (**B**) The surface potential of the Al-Si-Au calibration sample on a single line scan. (**C**) An illustration depicting the diode laser used for the illumination of both the (**D**) n-GaAs and (**E**) p-InAs samples.

S4. Treatment of the SUEM signal at negative time delays

To describe the temporal effect of the vacuum photo-induced contrast, we consider the time-of-flight (ToF) for a SE to overcome the influence of the SPV fields. The charge neutralization in the region under the laser can be approximated as a charged disc of radius '*r*'. The extent of the emitted fields (Δ) can be approximated as a function of distance '*z*' normal to the surface,

$$\Delta(z) = 1 - \frac{z}{\sqrt{r^2 + z^2}}. \qquad \text{Eq. (S1)}$$

The rise time of the vacuum contrast corresponds to the ToF to a distance of 1/*e* its peak value (τ_{TOF}). For a given SE of energy '*E*', this can be approximated as,

$$\tau_{\text{ToF}} \approx \sqrt{\frac{m_e r^2}{3E}}. \qquad \text{Eq. } (S2)$$

where m_e is the mass of an electron. SEs are emitted with a distribution of energies (*52*) such that the appearance of the vacuum contrast will occur approximately as the weighted average ToF over

the SE relevant energies. For a typical SE energy distribution and a beam radius of 100 μm, we calculate a distribution-weighted average ToF of τ_{ToF} = 65 ps.

Next, we develop a functional form for how electric field action over the ToF affects the time dependent SUEM signal. The SUEM signal is proportional to the change in the probability in detecting a SE when the pump in on relative to that when the pump in off. The probability of detecting an electron can be represented as the probability of the SE escaping from the surface P_{SS} multiplied by the probability and electron travels under the influence of the vacuum fields to the detector P_{VF}. The action of the pump laser perturbs these probabilities by ΔP_{SS} and ΔP_{VF}

$$\begin{aligned} R(t) &\propto \Delta P_{det,on}(t) \\ &= P_{SS,on}(t) P_{VF,on}(t) \\ &= \left(P_{SS,0} + \Delta P_{SS}(t)\right)\left(P_{VF,0} + \Delta P_{VF}(t)\right) \\ &= P_{SS,0}P_{VF,0} + \Delta P_{SS}(t)P_{SS,0} + \Delta P_{VF}(t)P_{VF,0} + \Delta P_{SS}(t)\Delta P_{VF}(t) \\ &\approx A\Delta P_{SS}(t) + B\Delta P_{VF}(t), \end{aligned}$$

Eq. (S3)

In Eq. (S3), in the last step, we recognize that $\Delta P_{SS} << P_{SS}$ and $\Delta P_{VF} << P_{VF}$ so the cross term can be ignored, and the time evolving components have been extracted from the time invariant background as is experimentally done. '*A*' and '*B*' are arbitrary constants, which represent the relative magnitude of SS and VF contrast respectively. In this pump-probe response the finite duration of the action of the pump on the probe electrons must be included between the material response $S(t)$ and the instrument response $Q(t)$ as

$$\begin{aligned} R(t) &= (S(t) * P_{det}(t)) \; * Q(t)) \\ &= ((S \; * (A\Delta P_{SS} + B\Delta P_{VF}) * Q) \\ &= A\big((S * \Delta P_{SS}) * Q\big) + B\big((S * \Delta P_{VF}) * Q\big) \end{aligned}$$

Eq. (S4)

In out SUEM experiment the relevant experimental time scales are τ_{SS} < 1 ps, τ_{ToF} = 65 ps and τ_{IRF} = 15 ps. The convolution of two normalizable functions approximately has the duration of the longer function. Therefore we can drop the shorter response function in the convolution.

$$R(t) \approx A(S * Q) + B(S * \Delta P_{VF}). \qquad \text{Eq. (S5)}$$

Instrument response function $Q(t)$ is Gaussian centered around t0, while the pump action through vacuum fields $P_{det}(t)$ increases monotonically and extends to negative times. Therefore we have developed a functional form for a SUEM instrument response.

$$R(t) \approx A\int_{-\infty}^{\infty} \frac{S(\tau)}{2\pi\tau_{\mathrm{IRF}}} \exp\left[\frac{-(t-\tau)^2}{2\tau_{\mathrm{IRF}}^2}\right] d\tau + B\int_{-\infty}^{\infty} \frac{S(\tau)H(t-\tau)}{T_{\mathrm{ToF}}} \exp\left[\frac{-(t-\tau)}{T_{\mathrm{ToF}}}\right] d\tau,$$

Eq. (S6)

S is the material response of interest, and *H* is the Heaviside step function. As seen, the detected signal can be represented as the sum of sub-surface and vacuum contrast effects with relative contribution coefficients *A* and *B*, respectively. The contribution from the sub-surface contrast is convolved with the instrument response function having a duration of 15 ps (the cross correlation of the pump and probe pulses), while the vacuum contribution is convolved with an exponential with a time constant τ_{ToF} of about 65 ps (the weighted ToF over the relevant SE energies).

The appearance of the contrast at negative time delays is inherent to the SUEM technique when low energy SEs are used to probe and when fields are generated by the pump laser. This complicates the data analysis but does not necessarily limit the time resolution of the experiment. Through least square fitting the material response ($S(t)$), can be extracted from the detected signal, ($R(t)$).

S5. Light-induced secondary electron loss and gain

The lock-in amplifier that processes the 1 MHz SUEM signal applies an arbitrary phase between the reference and input signal that obscures the absolute sign of the output signal. In order to the quantify the magnitude and sign of the change in SE emission, we compare our un-modulated 2 MHz SEM images with the pump laser on and off for p- and n-type InAs in Fig. S3 and Fig. S4, respectively.

As seen in Fig. S3, the p-InAs sample was exposed to a pump power of 312 μW, producing a peak laser fluence of 600 nJ cm^{-2}. With the pump-probe time delay was set to 30 ps, the signal was averaged in eight selected areas, shown in the insets of the figure. A dark spot appears in the middle of the image with the pump laser incident in Fig. S3A, showing a 3% decrease in SE yield from the same image with the pump laser off in Fig. S3B. The three regions surrounding the central laser spot on the other hand, exhibit a 4% increase in SE yield. We note that the four regions at the corners of the image show a negligible change in SE emission.

The decrease in signal for p-InAs going from pump off, Fig. S3B, to pump on, Fig. S3A, is attributed to the neutralization of the downward band edge curvature of a p-type material, halting the acceleration of SEs into the vacuum. The regions around the beam spot that became brighter are attributed to the negative photovoltage fields emitted from the central region where the pump beam is incident, repelling the SEs in the vacuum at short lengths above the sample.

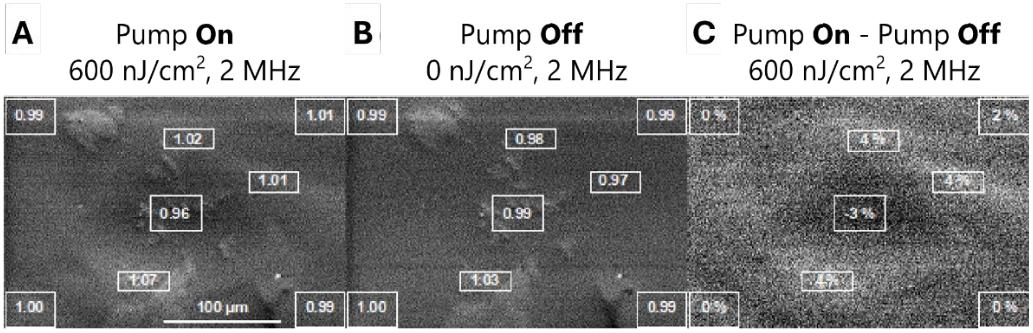

Figure S3. Pump on-off images obtained from the SUEM. SEM images of p-InAs acquired with a 312 µW of un-modulated 2 MHz pump power and a 2 MHz pulsed electron beam. The intensity of the signal was averaged in eight regions of interest (**A**) SEM images acquired with the optical pump was set to a time delay of 30 ps. Laser modulation was halted so that light was incident on the sample with every e-beam pulse. (**B**) SEM image with the optical pump blocked. (**C**) The SUEM image formed by subtraction of pump blocked from pump unblocked images. The signal intensity is indicated in the insets of images (A-B), and the percent change in signal is indicated in the inset of (C).

For n-InAs (MTI, LEC grown (100), Sn-doped, N_a = 3-10×10^{17} cm^{-3}), a dark contrast (~1% decrease) was observed at both positive and negative time delays, displayed in Fig. S4. For these experiments, we apply time delays of 107 ps (Fig. S4A) and -213 ps (Fig. S4B) at a pump power of 20 mW producing a peak laser fluence of 35 µJ cm^{-2}. This suggests that the contrast mechanism persists for longer than the 500 ns interval between pulses in n-InAs due to pulse accumulation within the sample. We note that with such long-lived carrier lifetimes, the picosecond dynamics could not be resolved through the stroboscopic SUEM technique for this material.

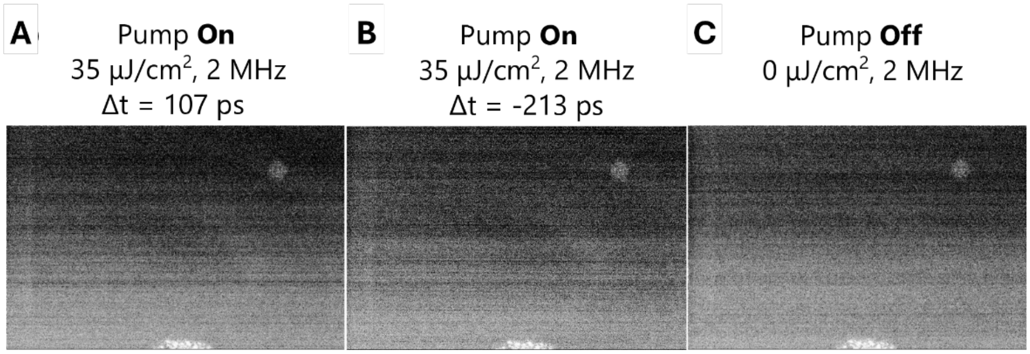

Figure S4. Pump-on images obtained from the SUEM at different time delays. SEM images of n-InAs acquired with a 20 mW of un-modulated pump power and a 2 MHz pulsed e-beam. SEM images acquired with a pump-probe time delay of (**A**) 107 ps and (**B**) -213 ps. (**C**) The SEM image with the optical pump blocked. Dark photo-induced SE contrast is observed at negative time delays, indicating that the contrast mechanism persists longer than 500 ns and pulse accumulation effects preclude resolving picosecond dynamics.

S6. Rutherford backscattering spectrometry

To study the composition of our GaAs and InAs single crystal substrates, a 2.3 MeV beam of He-4 ions with a spot diameter of nearly 2 mm was used to perform Rutherford Backscattering Spectrometry (RBS). The energy per channel was 1.367 keV ch^{-1}. In RBS, the impinging beam of energetic ions is backscattered from the the sample and gives information about their elemental composition. In Fig. S5, we show the RBS spectra and corresponding simulated spectrum for Si-doped GaAs and Zn-doped InAs single crystal substrates. SIMNRA program was used for simulation of experimental data (*53*). As shown in Fig. S5 A and B, a shoulder can be observed for both Ga and As (zoomed in the inset), close to each other, while a clear hump can be seen for In and As. The latter can be attributed due to distinct differences in their atomic mass. Furthermore, as can be seen in Fig. S5, no distinct feature has been observed for Si or Zn, suggesting very dilute concentration of dopants well below the detection limit of the technique. The ratio of atomic density obtained from the simulations shows a near-perfect 1:1 ratio for Ga:As and In:As respectively indicating high stoichiometric quality of the samples. Identifying this stoichiometry with a high confidence is crucial to this study because by this identification, we establish the vast differences in the bandgap, band-bending, etc., between n-GaAs and p-InAs, which further highlight the utility of our dynamical measurements that can probe materials with many orders of magnitude differences in surface states.

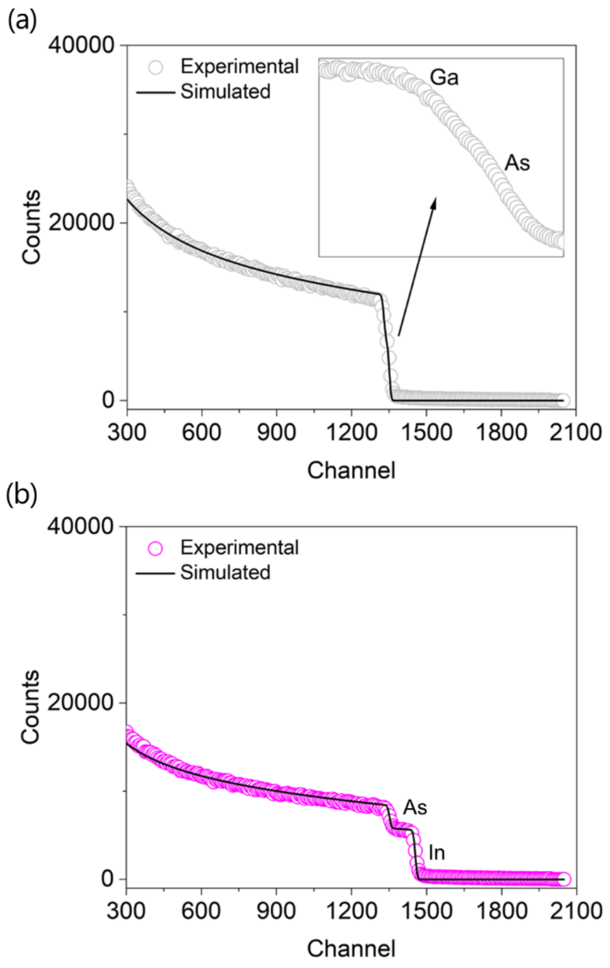

Figure S5. Rutherford Back-Scattering (RBS) data on the measured samples. RBS spectra and corresponding simulated spectrum for (**A**) Si-doped GaAs and (**B**) Zn-doped InAs single crystal substrates.

S7. Estimation of the electrostatic environment

As discussed in the main text, the edges of the probed region showed opposite contrast behavior than the sub-surface mechanism and was thus attributed to the vacuum-field mechanism. To calculate the impact of the generated charge on the trajectories of the electrons outside of the sample we consider the electric field due to a uniformly charged disc of radius R. Along the axis of the disc, the electric field is

$$E(r,z) = \frac{\sigma}{2\epsilon_0}\left(1 - \frac{z}{\sqrt{z^2 + R^2}}\right), \qquad \text{Eq. (S7)}$$

where ε_0 is the permittivity of free space, σ is the areal charge density of the disc, and z is the distance coordinate along the disc axis. In principle, the electric field is also a function of the perpendicular distance coordinates. However, for the distances >> R over which the electrons

travel, the disc is well approximated by a point charge with an isotropic field of magnitude given by Eq. (S7).

From the main text, we find a surface state density of 2×10^{13} cm^{-2} for n-GaAs and 5×10^{11} cm^{-2} for p-InAs. This can be compared with the fields present in the experiment. The vertical field is set mainly by the −300V applied to the sample compared with the grounded SEM tip, which is located a distance of 14.5 mm from the sample surface. This gives a vertical field of about 20,000 V m^{-1}. The lateral field determining the electron trajectory comes primarily from the +350V applied to the detector surface compared with the sample surface, yielding an estimate of 12,000 V m^{-1}.

All our measurements are over small spatial regions illuminated by the laser, with a spot size of 100 μm × 100 μm. Thus, despite the large surface charge densities mentioned above, the perturbation in the electric fields produced by the band bending at the surface is limited to small spatial distances. Particularly, at the detector, the electric fields due to the surface charges is minimal (<1 V m^{-1}). Thus, we adopt a simplified treatment of the problem, wherein our measurements reflect the impact to the electron trajectories during the initial impact (close to the surface) of the altered electric fields. In other words, our externally applied fields dominate in determining the overall electron trajectories, while our measured contrasts depend on our measurement's signal-to-noise ratio.

S8. Fluence saturation dependence in p-InAs and n-GaAs

As mentioned in the main text, the areal density of absorbed photons at saturation can be directly related to the surface trap state density for the semiconductors. Both p-InAs and n-GaAs exhibiting saturation behavior at fluences of 0.2 μmJ/cm^2, 8 μmJ/cm^2, respectively. Using the direct bandgap absorptivities of p-InAs and n-GaAs of 3×10^5 cm^{-1} (*38*) and 6×10^5 cm^{-1}, the photoexcitation is predicted to occur close to the surface. Accounting for the angles of incidence and refractive indices, an effective absorption depths of l = 30 nm and l = 15 nm are calculated for p-InAs and n-GaAs, respectively. The diffusivity of electrons is roughly D_e = 1000 cm^2 s^{-1} for p-InAs and D_h 200 cm^2 s^{-1} for n-GaAs. The relevant time scale (τ_D) for photoexcited electrons to diffuse to and from the surface layer can be approximated from

$$\tau_D = \frac{l^2}{D_e}, \qquad \text{Eq. (S8)}$$

We find the relevant time scale is approximately 1 ps in both semiconductors: far shorter than our instrument response of 14 ps. By 281 ps, when we probe the sample, most of the minority carriers have encountered the interface and have had the opportunity to become trapped by the surface potential. Thus, the areal density of absorbed photons at saturation can be directly related to the surface trap state density for the semiconductors. These trap state densities were found to be 5×10^{11} and 2×10^{13} cm^{-2} for p-InAs and n-GaAs, respectively.

S9. Measurement of beam profile

The laser was measured in the sample chamber at the point of focus with a beam profiler (Thorlabs BP109-IR2) mounted such that it was normal to the electron beam, seen in Fig. S6. This positioning was intended to capture the angle of incidence of the pump beam. Measurements with the beam profiler were performed immediately after SUEM experiments and the reflectivity of the p-polarized laser on the sample surface was measured to be less than 5%.

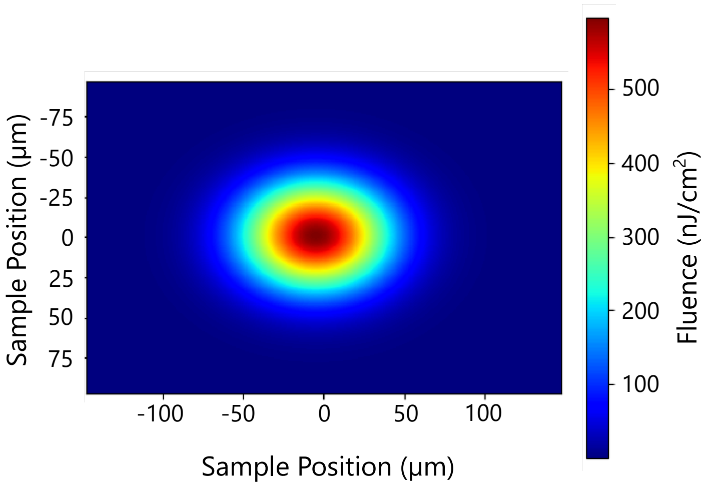

Figure S6. An example of a fluence profile. The fluence profile of the 25 μW 1 MHz laser beam at the focal point on the sample. The 1/*e* radii in the *x* and *y* dimensions were measured to be $w_x = 64$ μm and $w_y = 47$ μm, respectively. The peak fluence was measured to be 600 nJ cm^{-2}.

S10. Carrier diffusion and interfacial trapping model

As stated in the main text, prior work from our group (*33*) has shown that the material response is well reproduced by a modeling the 1D diffusion of charge carriers near a surface treated as a saturable adsorption boundary condition. As such, an area density of photoexcited carriers $\rho_C(t,z)$ is modeled to be rapidly injected into the system at time zero to prepare the initial carrier density. The SUEM signal is presumed to be proportional to the density of charge separated surface trap states,

$$S(t) \propto \rho_{\text{trap}}(t). \qquad \text{Eq. (S9)}$$

The traps form from a density of photoexcited charge carriers $\rho_{cc}(t,z)$ at $z = 0$ and provide an initial condition of charge carriers,

$$\rho_{\text{cc}}(0,z) = \frac{f}{h\nu} e^{-\alpha z}, \qquad \text{Eq. (S10)}$$

where f is laser fluence, α is attenuation coefficient, h is Planck's constant, and v is the frequency of light. The number of surface states ρ_{sat} is a material constant of interest, and it is important to note that the surface state are initially vacant,

$\rho_{\mathrm{trap}} = 0,$ Eq. (S11)

$\rho_{\mathrm{vacant}}(0) = \rho_{\mathrm{sat}}.$ Eq. (S12)

The charge carriers diffuse according to the Einstein diffusion model,

$$\frac{\partial \rho_{\mathrm{cc}}(t,z)}{\partial t} = D_{\mathrm{e}} \frac{\partial^2 \rho_{\mathrm{e}}(t,z)}{\partial z^2}. \qquad \text{Eq. (S13)}$$

The lateral gradients in carrier density in this experiment are much smaller than vertical gradients and are therefore not included in this model. At the interface, a Langmuir adsorption dynamic equilibrium exists between the free carriers at the surface and the vacant and occupied traps (*47*), representing a boundary condition. The equilibrium is re-established faster than diffusion can occur,

$$\rho_{\mathrm{cc}}(t,0) + \rho_{\mathrm{vacant}}(t) \overset{K}{\Leftrightarrow} \rho_{\mathrm{trap}}(t) \xrightarrow{1/\tau_R} 0\,. \qquad \text{Eq. (S14)}$$

The equilibrium constant K is defined as,

$$K = \frac{\rho_{\mathrm{trap}}(t)}{\rho_{\mathrm{cc}}(0,t)\left(\rho_{\mathrm{sat}} - \rho_{\mathrm{trap}}(t)\right)}. \qquad \text{Eq. (S15)}$$

The total density of free and trapped charge carriers that are conserved at a given time can be written as,

$$\rho_{\mathrm{total}}(t) = \rho_{\mathrm{cc}}(t,z) + \rho_{\mathrm{trap}}(t). \qquad \text{Eq. (S16)}$$

From Eq. S15, the density of free charge carriers at the surface can be reevaluated in between iterations of the diffusion model,

$$\rho_{\mathrm{cc}}(0,t) = \frac{1}{2K}\left(-1 - K\rho_{\mathrm{sat}} + K\rho_{\mathrm{total}}(t) - \sqrt{4K\rho_{\mathrm{total}}(t) + (1 - K\rho_{\mathrm{sat}} + K\rho_{\mathrm{total}}(t)^2)}\right)$$

Eq. (S17)

The 1D diffusion saturatable surface model (DSSM) was evaluated for the region of 0 um to 20 μm from the surface with a spatial grid of 330 nm. Time steps of 4 ps were used to sweep a time series spanning 0 to 10 ns. The charge carrier diffusion coefficients D_{cc} for n-GaAs and p-InAs were set to 200 and 1000 $\mathrm{cm^2\,s^{-1}}$, respectively (*54*). α for n-GaAs and p-InAs were set to 6.6×10^5 $\mathrm{cm^{-1}}$ and 3.3×10^5 $\mathrm{cm^{-1}}$ respectively. ρ_{sat} was fixed to the value determined in Figure 5 of the main text (2×10^{13} $\mathrm{cm^2}$ for n-GaAs and 5×10^{11} $\mathrm{cm^2}$ for p-InAs). Parameters τ_{R} and K were adjusted to best fit the time dependence and fluence dependence simultaneously. Once the fit parameters were determined ($\tau_{\mathrm{R}} = 5$ ns and $K = 0.5$ $\mathrm{cm^2}$), the full SUEM image time series could be analyzed. Fig. S7 shows the time-dependent SUEM signal and the model fits for both n-GaAs and p-InAs.

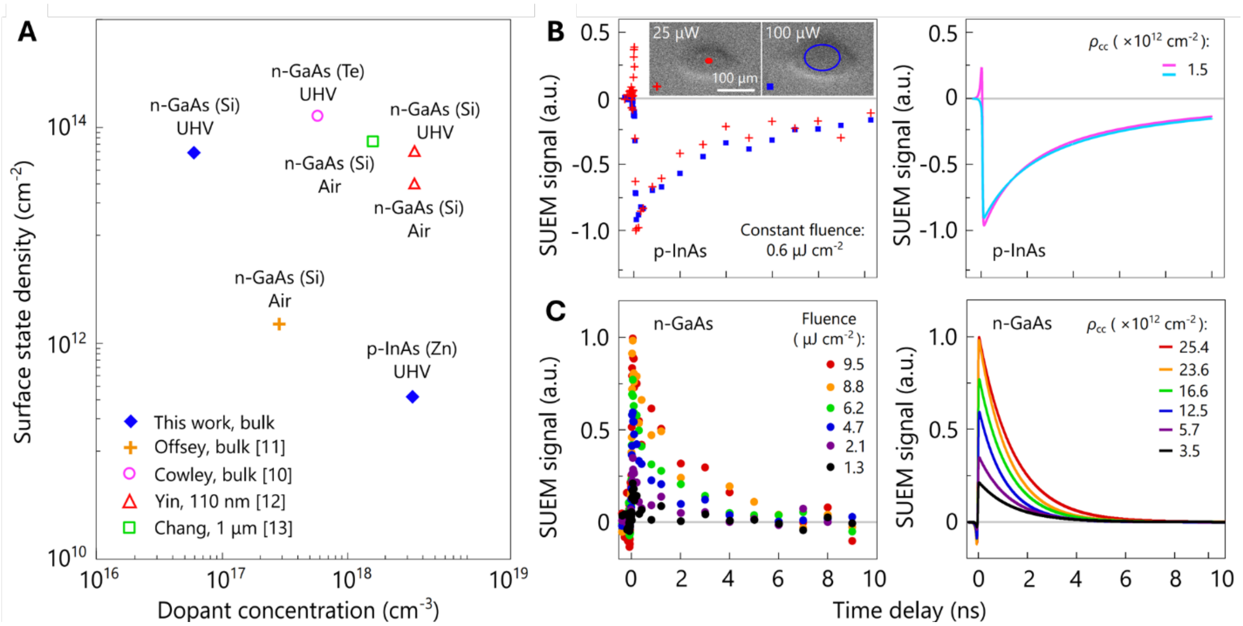

Figure S7. Comparison of dynamics in GaAS and InAs. (**A**) A benchmark plot displaying the various surface state densities with respect to dopant concentration found in the literature (*41-44*). The labels include the dopant type, the form factor of the sample in parentheses, and the measurement conditions, in air or ultra-high vacuum (UHV). The time-resolved SUEM signal extracted from (**B**) p-InAs and (**C**) n-GaAs. As shown in the main text, the red and blue elliptical regions with a 0.6 µJ cm^{-2} fluence were obtained with laser powers of 25 µW (red crosses) and 100 µW (blue squares) for p-InAs. The solid lines are fits to the instrument response function, Eq. S6.

Figure S8 presents the time evolution of carrier density in the DSSM of n-GaAs. At early times delays, the carriers diffuse towards the surface, which initially acts as an efficient sink producing charge separated carriers. The occupied traps modify the surface potential and the SUEM signal evolves in less than 80 ps accelerated by the high initial carrier density $\langle \rho_{cc}(z,0)\rangle > \rho_{sat}$. After the initial saturation the free carriers have largely diffused away from the surface and the traps decay both through recombination and detrapping.

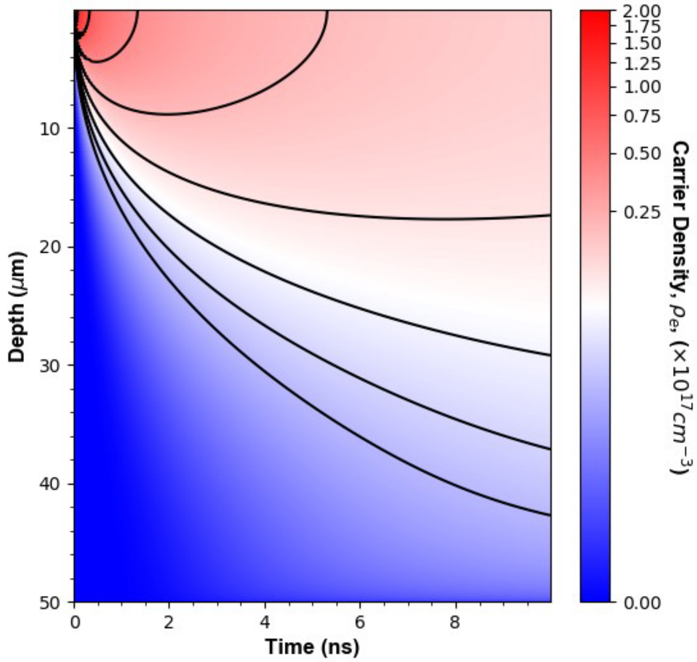

Figure S8. Simulated carrier diffusion data. Simulated free carrier diffusion of $\rho_{CC}(z,t)$ at the n-GaAs surface after excition to an 26×10^{12} cm^{-2}. The model parameters were the same as those used in Fig. S7C listed in the main text.

S11. Comparison of SUEM signals at different laser powers

In this section, we compare the SUEM signals on both n-GaAs and p-InAs as a function of power and time delay. The time series for both n-GaAs and p-InAs from -200 ps to 10,000 ps at two different laser fluences is given in Fig. S9.

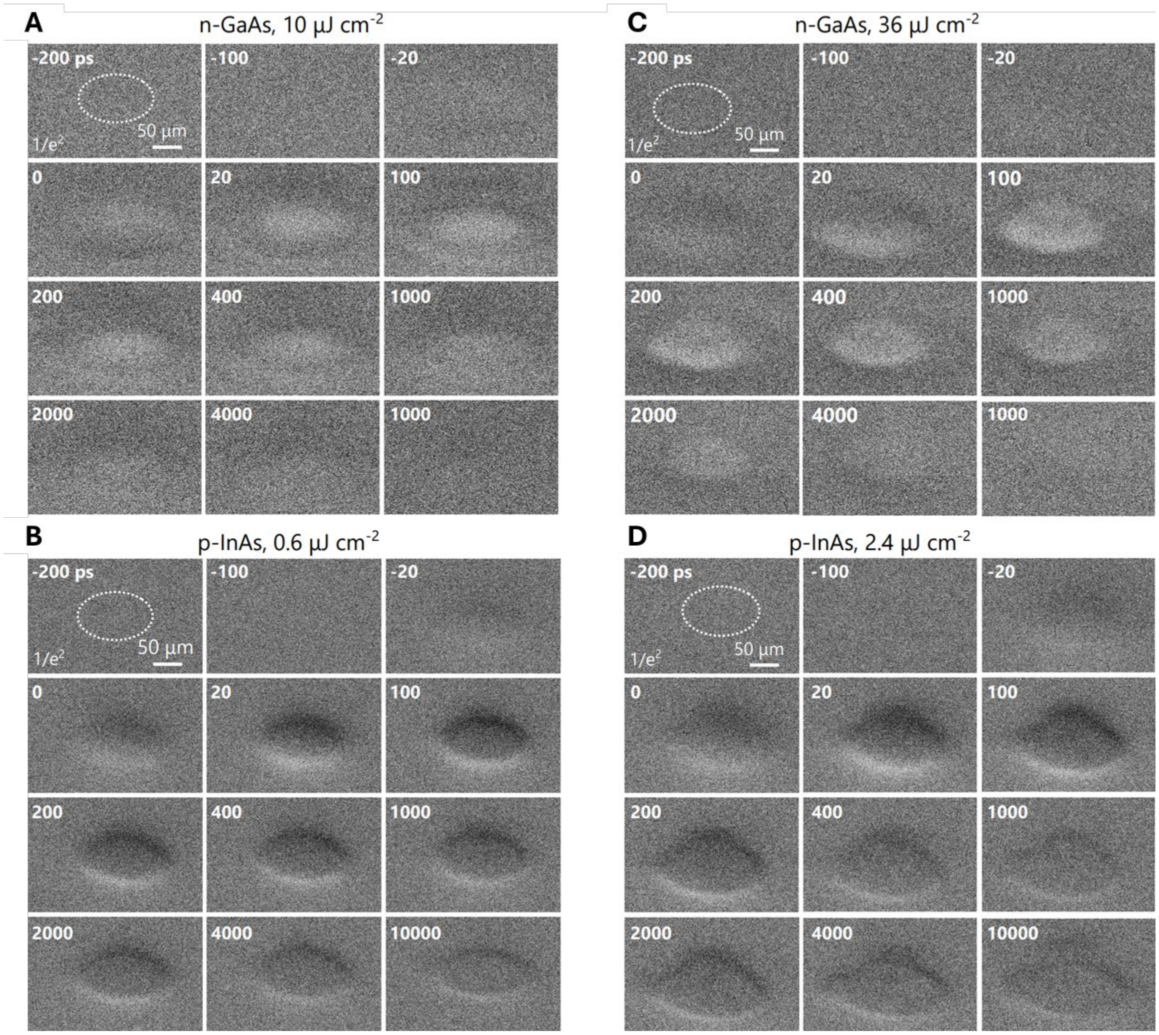

Figure S9. SUEM image time series acquired with two different laser powers. Data on (**A,C**) n-GaAs and (**B,D**) p-InAs. The peak fluences are shown in the time series headers, while the beam $1/e^2$ radii are indicated with the dotted circles. The time delay (in ps) is provided in the top left inset of each image.

S12. Analysis of second moment contrast

Here, we analyze the effect of the second moment contrast on our implementation of the SUEM technique. The second moment is used to bound the probability that our SUEM signal fluctuates far from its mean via determining its moments. The second moment of the SUEM signal is calculated as,

$$\langle R^2\rangle(t) = \frac{\sum_{i,j}(x_i^2 + y_j^2) I(x_i, y_i, t)}{\sum_{i,j} I(x_i, y_i, t)} \qquad \text{Eq. (S18)}$$

for the magnitude of our signal $\langle R^2\rangle$, and,

$$\langle X^2\rangle/\langle Y^2\rangle(t) = \frac{\sum_{i,j}(x_i^2)I(x_i,y_i,t)}{\sum_{i,j}(y_i^2)I(x_i,y_i,t)} \qquad \text{Eq. (S19)}$$

for the squared elliptical aspect ratio of our signal. The calculations for both are shown in Fig. S10.

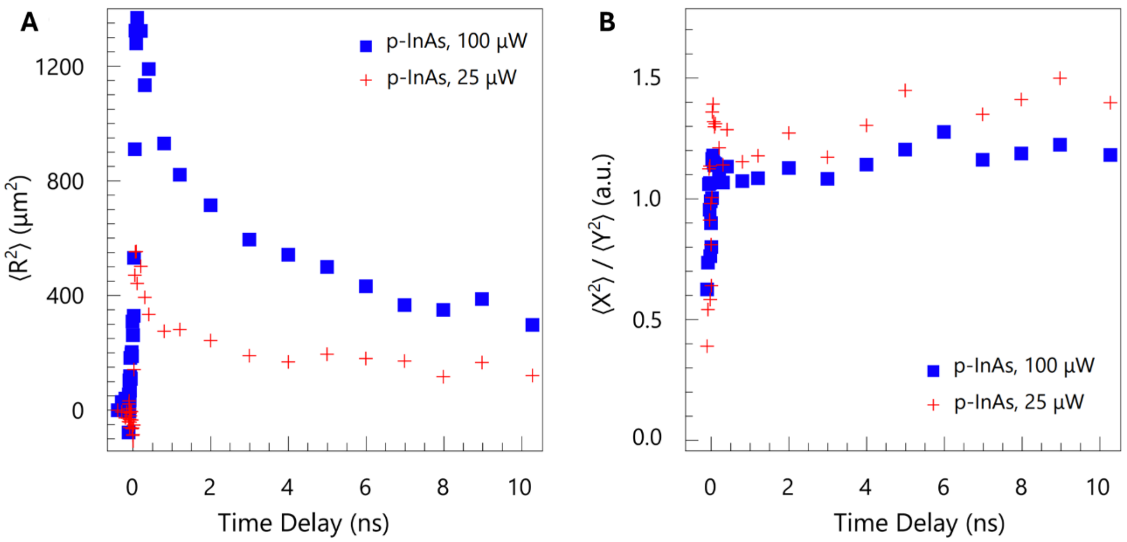

Figure S10. The calculated second moment of the SUEM signal. Data acquired with laser powers of 25 μW (red crosses) and 100 μW (blue squares). The calculations are shown for the (**A**) magnitude and (**B**) squared elliptical aspect ratio of our SUEM signal.

Figure S10A demonstrates how the magnitude of our SUEM signal rapidly expands, reaching a peak moment of 500 μm^2 with the 25 μW laser, and 1300 μm^2 with the 100 μW laser at a time delay of 80 ps. After 80 ps, however, the second moment contracts to roughly one fourth of its peak value by 10 ns. In previous reports (*27*), the second moment has been used as a metric for the electron and hole spatial distribution. The rapid increase of the second moment was taken to evidence the so called 'super diffusion' (*29, 30*). However, from Fig. S9 we see that spatial distribution is dependent on the power and focus of the laser beam.

To further demonstrate how the spatial distribution is dependent on the power and focus of the laser, the squared elliptical aspect ratio of the SUEM signal was calculated with Eq. S19 and is shown in Fig. S10B. We note that the circular signal would have an aspect ratio of unity. The squared aspect ratio rapidly increases to between 1.1 and 1.4 at time zero and increases slightly at later time. If the expansion signal were attributed to a lateral diffusive process, the aspect ratio is expected to decrease because diffusion would be more rapid along the short elliptical axis due to a correspondingly greater concentration gradient. Instead, the squared aspect ratio parallels the aspect ratio of the laser spot of (w_x/w_y = 1.36). This observation indicates that that lateral 'super

diffusion' is not likely in this case and that a vertical diffusion and saturation phenomena can reconcile the data. Consequently, we do not believe that hot carriers have an effect in these studies.

REFERENCES AND NOTES

1. Z. D. Blount, R. E. Lenski, J. B. Losos, Contingency and determinism in evolution: Replaying life's tape. *Science* **362**, eaam5979 (2018).

2. P. F. Colosimo, K. E. Hosemann, S. Balabhadra, G. Villarreal Jr, M. Dickson, J. Grimwood, J. Schmutz, R. M. Myers, D. Schluter, D. M. Kingsley, Widespread parallel evolution in sticklebacks by repeated fixation of ectodysplasin alleles. *Science* **307**, 1928–1933 (2005).

3. D. L. Stern, The genetic causes of convergent evolution. *Nat. Rev. Genet.* **14**, 751–764 (2013).

4. D. M. Weinreich, N. F. Delaney, M. A. DePristo, D. L. Hartl, Darwinian evolution can follow only very few mutational paths to fitter proteins. *Science* **312**, 111–114 (2006).

5. S. J. Gould, *Wonderful Life—The Burgess Shale and the Nature of History* (W. W. Norton and Company, 1990).

6. S. C. Morris, Evolution: Like any other science it is predictable. *Philos. Trans. R. Soc. B: Biol. Sci.* **365**, 133–145 (2010).

7. J. B. Losos, *Lizards in an Evolutionary Tree: Ecology and Adaptive Radiation of Anoles* (University of California Press, 2011), vol. **10**.

8. J. Arendt, D. Reznick, Convergence and parallelism reconsidered: What have we learned about the genetics of adaptation? *Trends Ecol. Evol.* **23**, 26–32 (2008).

9. J. B. Losos, *Improbable Destinies: How Predictable Is Evolution?* (Penguin, 2017).

10. T. van der Valk, P. Pečnerová, D. Díez-del-Molino, A. Bergström, J. Oppenheimer, S. Hartmann, G. Xenikoudakis, J. A. Thomas, M. Dehasque, E. Sağlıcan, F. R. Fidan, I. Barnes, S. Liu, M. Somel, P. D. Heintzman, P. Nikolskiy, B. Shapiro, P. Skoglund, M. Hofreiter, A. M. Lister, A. Götherström, L. Dalén, Million-year-old DNA sheds light on the genomic history of mammoths. *Nature* **591**, 265–269 (2021).

11. G. J. Vermeij, Historical contingency and the purported uniqueness of evolutionary innovations. *Proc. Natl. Acad. Sci. U.S.A.* **103**, 1804–1809 (2006).

12. J. R. Meyer, D. T. Dobias, J. S. Weitz, J. E. Barrick, R. T. Quick, R. E. Lenski, Repeatability and contingency in the evolution of a key innovation in phage lambda. *Science* **335**, 428–432 (2012).

13. J. Plucain, T. Hindré, M. Le Gac, O. Tenaillon, S. Cruveiller, C. Médigue, N. Leiby, W. R. Harcombe, C. J. Marx, R. E. Lenski, D. Schneider, Epistasis and allele specificity in the emergence of a stable polymorphism in *Escherichia coli*. *Science* **343**, 1366–1369 (2014).

14. M. Travisano, J. A. Mongold, A. F. Bennett, R. E. Lenski, Experimental tests of the roles of adaptation, chance, and history in Evolution. *Science* **267**, 87–90 (1995).

15. Z. Gompert, F. J. Messina, Genomic evidence that resource-based trade-offs limit host-range expansion in a seed beetle. *Evolution* **70**, 1249–1264 (2016).

16. W. R. Rice, E. E. Hostert, Laboratory Experiments on speciation: What have we learned in 40 years? *Evolution* **47**, 1637–1653 (1993).

17. M. Bosse, L. G. Spurgin, V. N. Laine, E. F. Cole, J. A. Firth, P. Gienapp, A. G. Gosler, K. McMahon, J. Poissant, I. Verhagen, M. A. M. Groenen, K. Van Oers, B. C. Sheldon, M. E. Visser, J. Slate, Recent natural selection causes adaptive evolution of an avian polygenic trait. *Science* **358**, 365–368 (2017).

18. A. Charmantier, R. H. McCleery, L. R. Cole, C. Perrins, L. E. B. Kruuk, B. C. Sheldon, Adaptive phenotypic plasticity in response to climate change in a wild bird population. *Science* **320**, 800–803 (2008).

19. P. R. Grant, B. R. Grant, Evolution of character displacement in Darwin's finches. *Science* **313**, 224–226 (2006).

20. P. R. Grant, B. R. Grant, Unpredictable evolution in a 30-year study of Darwin's finches. *Science* **296**, 707–711 (2002).

21. I. Hanski, Metapopulation dynamics. *Nature* **396**, 41–49 (1998).

22. H. B. D. Kettlewell, Selection experiments on industrial melanism in the Lepidoptera. *Heredity* **9**, 323–342 (1955).

23. M. C. Singer, C. Parmesan, Lethal trap created by adaptive evolutionary response to an exotic resource. *Nature* **557**, 238–241 (2018).

24. D. A. Marques, F. C. Jones, D. Palma, D. M. Kingsley, T. E. Reimchen, Experimental evidence for rapid genomic adaptation to a new niche in an adaptive radiation. *Nat. Ecol. Evol.* **2**, 1128–1138 (2018).

25. T. E. Reimchen, Predator-induced cyclical changes in lateral plate frequencies of *Gasterosteus*. *Behaviour* **132**, 1079–1094 (1995).

26. T. E. Reimchen, P. Nosil, Temporal variation in divergent selection on spine number in threespine stickleback. *Evolution* **56**, 2472–2483 (2002).

27. D. N. Reznick, H. Bryga, Life-history evolution in guppies (*Poecilia reticulata*): 1. Phenotypic and genetic changes in an introduction experiment. *Evolution* **41**, 1370–1385 (1987).

28. S. E. Johnston, J. Gratten, C. Berenos, J. G. Pilkington, T. H. Clutton-Brock, J. M. Pemberton, J. Slate, Life history trade-offs at a single locus maintain sexually selected genetic variation. *Nature* **502**, 93–95 (2013).

29. A. O. Bergland, E. L. Behrman, K. R. O'Brien, P. S. Schmidt, D. A. Petrov, Genomic evidence of rapid and stable adaptive oscillations over seasonal time scales in *Drosophila*. *PLOS Genet.* **10**, e1004775 (2014).

30. L. E. B. Kruuk, J. Slate, J. M. Pemberton, S. Brotherstone, F. Guinness, T. Clutton-Brock, Antler size in red deer: Heritability and selection but no evolution. *Evolution* **56**, 1683–1695 (2002).

31. R. D. H. Barrett, D. Schluter, Adaptation from standing genetic variation. *Trends Ecol. Evol.* **23**, 38–44 (2008).

32. G. L. Conte, M. E. Arnegard, C. L. Peichel, D. Schluter, The probability of genetic parallelism and convergence in natural populations. *Proc. R. Soc. B: Biol. Sci.* **279**, 5039–5047 (2012).

33. M. Bohutínská, C. L. Peichel, Divergence time shapes gene reuse during repeated adaptation. *Trends Ecol. Evol.* **39**, 396–407 (2024).

34. Z. D. Blount, C. Z. Borland, R. E. Lenski, Historical contingency and the evolution of a key innovation in an experimental population of *Escherichia coli*. *Proc. Natl. Acad. Sci.* **105**, 7899–7906 (2008).

35. S. Kryazhimskiy, D. P. Rice, E. R. Jerison, M. M. Desai, Global epistasis makes adaptation predictable despite sequence-level stochasticity. *Science* **344**, 1519–1522 (2014).

36. R. M. Varney, D. I. Speiser, J. T. Cannon, M. A. Aguilar, D. J. Eernisse, T. H. Oakley, A morphological basis for path-dependent evolution of visual systems. *Science* **383**, 983–987 (2024).

37. S. Yeaman, Local adaptation by alleles of small effect. *Am. Natural.* **186**, S74–S89 (2015).

38. J. F. Storz, Causes of molecular convergence and parallelism in protein evolution. *Nat. Rev. Genet.* **17**, 239–250 (2016).

39. B. J. Crespi, C. P. Sandoval, Phylogenetic evidence for the evolution of ecological specialization in Timema walking-sticks. *J. Evol. Biol.* **13**, 249–262 (2000).

40. A. A. Comeault, S. M. Flaxman, R. Riesch, E. Curran, V. Soria-Carrasco, Z. Gompert, T. E. Farkas, M. Muschick, T. L. Parchman, T. Schwander, J. Slate, P. Nosil, Selection on a genetic polymorphism counteracts ecological speciation in a stick insect. *Curr. Biol.* **25**, 1975–1981 (2015).

41. P. Nosil, Divergent host plant adaptation and reproductive isolation between ecotypes of *Timema cristinae* walking sticks. *Am. Nat.* **169**, 151–162 (2007).

42. P. Nosil, B. J. Crespi, Experimental evidence that predation promotes divergence in adaptive radiation. *Proc. Natl. Acad. Sci. U.S.A.* **103**, 9090–9095 (2006).

43. C. P. Sandoval, Differential visual predation on morphs of *Timema cristinae* (Phasmatodeae:Timemidae) and its consequences for host range. *Biol. J. Linn. Soc.* **52**, 341–356 (1994).

44. P. Nosil, B. J. Crespi, C. P. Sandoval, Host-plant adaptation drives the parallel evolution of reproductive isolation. *Nature* **417**, 440–443 (2002).

45. P. Nosil, R. Villoutreix, C. F. de Carvalho, T. E. Farkas, V. Soria-Carrasco, J. L. Feder, B. J. Crespi, Z. Gompert, Natural selection and the predictability of evolution in Timema stick insects. *Science* **359**, 765–770 (2018).

46. C. P. Sandoval, The effects of the relative geographic scales of gene flow and selection on morph frequencies in the walking-stick *Timema cristinae*. *Evolution* **48**, 1866–1879 (1994).

47. D. Lindtke, K. Lucek, V. Soria-Carrasco, R. Villoutreix, T. E. Farkas, R. Riesch, S. R. Dennis, Z. Gompert, P. Nosil, Long-term balancing selection on chromosomal variants associated with crypsis in a stick insect. *Mol. Ecol.* **26**, 6189–6205 (2017).

48. A. B. Bond, The evolution of color polymorphism: Crypticity, searching images, and apostatic selection. *Annu. Rev. Ecol. Evol. Syst.* **38**, 489–514 (2007).

49. A. B. Bond, A. C. Kamil, Visual predators select for crypticity and polymorphism in virtual prey. *Nature* **415**, 609–613 (2002).

50. J. A. Allen, B. Clarke, Evidence for apostatic selection by wild passerines. *Nature* **220**, 501–502 (1968).

51. J. A. Allen, Further evidence for apostatic selection by wild passerine birds—9:1 experiments. *Heredity* **36**, 173–180 (1976).

52. C. Sandoval, Persistence of a walking-stick population (Phasmatoptera: Timematodea) after a wildfire. *Southw. Natural.* **45**, 123–127 (2000).

53. P. Nosil, B. J. Crespi, C. P. Sandoval, Reproductive isolation driven by the combined effects of ecological adaptation and reinforcement. *Proc. R. Soc. Lond. B: Biol. Sci.* **270**, 1911–1918 (2003).

54. P. Nosil, *Ecological Speciation* (Oxford Univ. Press, 2012).

55. R. Villoutreix, C. F. de Carvalho, V. Soria-Carrasco, D. Lindtke, M. De-la-Mora, M. Muschick, J. L. Feder, T. L. Parchman, Z. Gompert, P. Nosil, Large-scale mutation in the evolution of a gene complex for cryptic coloration. *Science* **369**, 460–466 (2020).

56. R. Riesch, M. Muschick, D. Lindtke, R. Villoutreix, A. A. Comeault, T. E. Farkas, K. Lucek, E. Hellen, V. Soria-Carrasco, S. R. Dennis, C. F. De Carvalho, R. J. Safran, C. P. Sandoval, J. Feder, R. Gries, B. J. Crespi, G. Gries, Z. Gompert, P. Nosil, Transitions between phases of genomic differentiation during stick-insect speciation. *Nat. Ecol. Evol.* **1**, 82 (2017).

57. R. C. Lewontin, A general method for investigating the equilibrium of gene frequency in a population. *Genetics* **43**, 419–434 (1958).

58. L.-M. Chevin, Z. Gompert, P. Nosil, Frequency dependence and the predictability of evolution in a changing environment. *Evol. Lett.* **6**, 21–33 (2022).

59. J. K. Goldberg, C. M. Lively, S. R. Sternlieb, G. Pintel, J. D. Hare, M. B. Morrissey, L. F. Delph, Herbivore-mediated negative frequency-dependent selection underlies a trichome dimorphism in nature. *Evol. Lett.* **4**, 83–90 (2020).

60. D. I. Bolnick, P. Nosil, Natural selection in populations subject to a migration load. *Evolution* **61**, 2229–2243 (2007).

61. E. I. Svensson, J. Abbott, R. Härdling, Female polymorphism, frequency dependence, and rapid evolutionary dynamics in natural populations. *Am. Nat.* **165**, 567–576 (2005).

62. E. I. Svensson, T. Connallon, How frequency-dependent selection affects population fitness, maladaptation and evolutionary rescue. *Evol. Appl.* **12**, 1243–1258 (2019).

63. P. Jay, T. G. Aubier, M. Joron, The interplay of local adaptation and gene flow may lead to the formation of supergenes. *Mol. Ecol.* e17297 (2024).

64. P. Nosil, Reproductive isolation caused by visual predation on migrants between divergent environments. *Proc. R. Soc. Lond. B: Biol. Sci.* **271**, 1521–1528 (2004).

65. Z. Gompert, A. A. Comeault, T. E. Farkas, J. L. Feder, T. L. Parchman, C. A. Buerkle, P. Nosil, Experimental evidence for ecological selection on genome variation in the wild. *Ecol. Lett.* **17**, 369–379 (2014).

66. Z. Gompert, J. L. Feder, P. Nosil, The short-term, genome-wide effects of indirect selection deserve study: A response to Charlesworth and Jensen (2022). *Mol. Ecol.* **31**, 4444–4450 (2022).

67. V. Soria-Carrasco, Z. Gompert, A. Comeault, T. E. Farkas, T. L. Parchman, J. S. Johnston, C. A. Buerkle, J. L. Feder, J. Bast, T. Schwander, S. P. Egan, B. J. Crespi, P. Nosil, Stick insect genomes reveal natural selection's role in parallel speciation. *Science* **344**, 738–42 (2014).

68. S. Chaturvedi, Z. Gompert, J. L. Feder, O. G. Osborne, M. Muschick, R. Riesch, V. Soria-Carrasco, P. Nosil, Climatic similarity and genomic background shape the extent of parallel adaptation in *Timema* stick insects. *Nat. Ecol. Evol.* **6**, 1952–1964 (2022).

69. L. S. Zamorano, Z. Gompert, E. A. Fronhofer, J. L. Feder, P. Nosil, A stabilizing eco-evolutionary feedback loop in the wild. *Curr. Biol.* **33**, 3272–3278.e3 (2023).

70. A. A. Comeault, C. Ferreira, S. Dennis, V. Soria-Carrasco, P. Nosil, Color phenotypes are under similar genetic control in two distantly related species of *Timema* stick insect. *Evolution* **70**, 1283–1296 (2016).

71. C. P. Sandoval, P. Nosil, Counteracting selective regimes and host preference evolution in ecotypes of two species of walking-sticks. *Evolution* **59**, 2405–2413 (2005).

72. P. Nosil, V. Soria-Carrasco, R. Villoutreix, M. De-la-Mora, C. F. de Carvalho, T. Parchman, J. L. Feder, Z. Gompert, Complex evolutionary processes maintain an ancient chromosomal inversion. *Proc. Natl. Acad. Sci. U.S.A.* **120**, e2300673120 (2023).

73. S. Wright, The shifting balance theory and macroevolution. *Annu. Rev. Genet.* **16**, 1–20 (1982).

74. Y. F. Chan, M. E. Marks, F. C. Jones, G. Villarreal, M. D. Shapiro, S. D. Brady, A. M. Southwick, D. M. Absher, J. Grimwood, J. Schmutz, R. M. Myers, D. Petrov, B. Jónsson, D. Schluter, M. A. Bell, D. M. Kingsley, Adaptive evolution of pelvic reduction in sticklebacks by recurrent deletion of a Pitx1 enhancer. *Science* **327**, 302–305 (2010).

75. K. T. Xie, G. Wang, A. C. Thompson, J. I. Wucherpfennig, T. E. Reimchen, A. D. C. MacColl, D. Schluter, M. A. Bell, K. M. Vasquez, D. M. Kingsley, DNA fragility in the parallel evolution of pelvic reduction in stickleback fish. *Science* **363**, 81–84 (2019).

76. K.-W. Kim, R. De-Kayne, I. J. Gordon, K. S. Omufwoko, D. J. Martins, R. Ffrench-Constant, S. H. Martin, Stepwise evolution of a butterfly supergene via duplication and inversion. *Philosop. Trans. R. Soc. B: Biol. Sci.* **377**, 20210207 (2022).

77. D. J. Funk, Isolating a role for natural selection in speciation: Host adaptation and sexual isolation in *Neochlamisus bebbianae* leaf beetles. *Evolution* **52**, 1744–1759 (1998).

78. D. Schluter, Evidence for ecological speciation and its alternative. *Science* **323**, 737–741 (2009).

79. D. Schluter, *The Ecology of Adaptive Radiation* (Oxford Univ. Press, 2000).

80. P. Nosil, T. H. Vines, D. J. Funk, Reproductive isolation caused by natural selection against immigrants from divergent habitats. *Evolution* **59**, 705–719 (2005).

81. P. Nosil, B. J. Crespi, C. P. Sandoval, M. Kirkpatrick, Migration and the genetic covariance between habitat preference and performance. *Am. Nat.* **167**, E66–E78 (2006).

82. P. Nosil, C. P. Sandoval, B. J. Crespi, The evolution of host preference in allopatric vs. parapatric populations of *Timema cristinae* walking-sticks. *J. Evol. Biol.* **19**, 929–942 (2006).

83. P. Nosil, J. L. Feder, S. M. Flaxman, Z. Gompert, Tipping points in the dynamics of speciation. *Nat. Ecol. Evol.* **1**, 1 (2017).

84. M. Scheffer, S. R. Carpenter, T. M. Lenton, J. Bascompte, W. Brock, V. Dakos, J. Van de Koppel, I. A. Van de Leemput, S. A. Levin, E. H. Van Nes, M. Pascual, Anticipating critical transitions. *Science* **338**, 344–348 (2012).

85. R. C. Lewontin, *The Genetic Basis of Evolutionary Change* (Columbia Univ. Press, 1974).

86. P. Nosil, Z. Gompert, T. E. Farkas, A. A. Comeault, J. L. Feder, C. S. Buerkle, T. L. Parchman, Genomic consequences of multiple speciation processes in a stick insect. *Proc. R. Soc. B: Biol. Sci.* **279**, 5058–5065 (2012).

87. J. Armstrong, G. Hickey, M. Diekhans, I. T. Fiddes, A. M. Novak, A. Deran, Q. Fang, D. Xie, S. Feng, J. Stiller, D. Genereux, J. Johnson, V. D. Marinescu, J. Alföldi, R. S. Harris, K. Lindblad-Toh, D.

Haussler, E. Karlsson, E. D. Jarvis, G. Zhang, B. Paten, Progressive Cactus is a multiple-genome aligner for the thousand-genome era. *Nature* **587**, 246–251 (2020).

88. B. Paten, D. Earl, N. Nguyen, M. Diekhans, D. Zerbino, D. Haussler, Cactus: Algorithms for genome multiple sequence alignment. *Genome Res.* **21**, 1512–1528 (2011).

89. A. F. Smit, Repeat-Masker Open-3.0 (2004); www.repeatmasker.org.

90. K. Krasheninnikova, M. Diekhans, J. Armstrong, A. Dievskii, B. Paten, S. O'Brien, HalSynteny: A fast, easy-to-use conserved synteny block construction method for multiple whole-genome alignments. *GigaScience* **9**, giaa047 (2020).

91. H. Li, R. Durbin, Fast and accurate short read alignment with Burrows-Wheeler transform. *Bioinformatics* **25**, 1754–1760 (2009).

92. H. Li, B. Handsaker, A. Wysoker, T. Fennell, J. Ruan, N. Homer, G. Marth, G. Abecasis, R. Durbin, The sequence alignment/map format and SAMtools. *Bioinformatics* **25**, 2078–2079 (2009).

93. X. Zhou, M. Stephens, Genome-wide efficient mixed-model analysis for association studies. *Nat. Genet.* **44**, 821–824 (2012).

94. H. Li, P. Ralph, Local PCA shows how the effect of population structure differs along the genome. *Genetics* **211**, 289–304 (2019).

95. M. Todesco, G. L. Owens, N. Bercovich, J. S. Légaré, S. Soudi, D. O. Burge, K. Huang, K. L. Ostevik, E. B. M. Drummond, I. Imerovski, K. Lande, M. A. Pascual-Robles, M. Nanavati, M. Jahani, W. Cheung, S. E. Staton, S. Muños, R. Nielsen, L. A. Donovan, J. M. Burke, S. Yeaman, L. H. Rieseberg, Massive haplotypes underlie ecotypic differentiation in sunflowers. *Nature* **584**, 602–607 (2020).

96. W. J. Ewens, *Mathematical Population Genetics I. Theoretical Introduction*, Interdisciplinary Applied Mathematics (Springer, 2004).

97. S. Wright, *Evolution and the Genetics of Populations: A Treatise in Four, Volume 4: Variability Within and Among Natural Populations* (University of Chicago Press, 1978).

98. M. Plummer, rjags: Bayesian graphical models using MCMC, R package version 4-14 (2023); https://CRAN.R-project.org/package=rjags.

99. M. Plummer, N. Best, K. Cowles, K. Vines, CODA: Convergence diagnosis and output analysis for MCMC. *R News* **6**, 7–11 (2006).

100. R. M. Neal, "MCMC using Hamiltonian dynamics" in *Handbook of Markov Chain Monte Carlo* (CRC Press 2011), p. 113–160.

101. Stan Development Team, RStan: The R interface to Stan, R package version 2.21.8 (2023); https://mc-stan.org/.

102. M. D. Hoffman, A. Gelman, The No-U-Turn sampler: Adaptively setting path lengths in Hamiltonian Monte Carlo. *J. Mach. Learn. Res.* **15**, 1593–1623 (2014).

103. Z. Gompert, S. M. Flaxman, J. L. Feder, L.-M. Chevin, P. Nosil, Laplace's demon in biology: Models of evolutionary prediction. *Evolution* **76**, 2794–2810 (2022).

104. S. Rice, *Evolutionary Theory: Mathematical and Conceptual Foundations* (Sinauer Associates, 2004).

105. A. Vehtari, A. Gelman, J. Gabry, Practical Bayesian model evaluation using leave-one-out cross-validation and WAIC. *Statist. Comput.* **27**, 1413–1432 (2017).

106. N. H. Putnam, B. L. O'Connell, J. C. Stites, B. J. Rice, M. Blanchette, R. Calef, C. J. Troll, A. Fields, P. D. Hartley, C. W. Sugnet, D. Haussler, D. S. Rokhsar, R. E. Green, Chromosome-scale shotgun assembly using an in vitro method for long-range linkage. *Genome Res.* **26**, 342–350 (2016).

107. E. Lieberman-Aiden, N. L. Van Berkum, L. Williams, M. Imakaev, T. Ragoczy, A. Telling, I. Amit, B. R. Lajoie, P. J. Sabo, M. O. Dorschner, R. Sandstrom, B. Bernstein, M. A. Bender, M. Groudine, A. Gnirke, J. Stamatoyannopoulos, L. A. Mirny, E. S. Lander, J. Dekker, Comprehensive mapping of long-range interactions reveals folding principles of the human genome. *Science* **326**, 289–293 (2009).

108. D. Harte, HiddenMarkov: Hidden Markov Models, Statistics Research Associates, Wellington, R package version 1.8-13 (2021); www.statsresearch.co.nz/dsh/sslib/.

109. L. E. Baum, T. Petrie, G. Soules, N. Weiss, A maximization technique occurring in the statistical analysis of probabilistic functions of Markov chains. *Ann. Math. Stat.* **41**, 164–171 (1970).

110. G. D. Forney, The Viterbi algorithm. *Proc. IEEE* **61**, 268–278 (1973).

111. J. Ruan, H. Li, Fast and accurate long-read assembly with wtdbg2. *Nat. Methods* **17**, 155–158 (2020).

112. D. R. Laetsch, M. L. Blaxter, BlobTools: Interrogation of genome assemblies. *F1000Res.* **6**, 1287 (2017).

113. D. Guan, S. A. McCarthy, J. Wood, K. Howe, Y. Wang, R. Rubin Identifying and removing haplotypic duplication in primary genome assemblies. *Bioinformatics* **36**, 2896–2898 (2020).
